# Short‐Term, Cycle‐Synchronized Adjunctive Therapy With Crocus Total Glucosides Tablets for Cardiac Protection Against Cancer Therapy‐Related Cardiac Dysfunction in Breast Cancer Patients: A Randomized, Double‐Blind, Placebo‐Controlled Trial

**DOI:** 10.1002/mco2.70780

**Published:** 2026-05-28

**Authors:** Xiaoling Liu, Mengmeng Li, Wenwen Song, Yu Zhang, Wuyun Bao, Chaoyu Liu, Yuan Zhang, Quande Liu, Cheng Zhang, Yun Zhang, Li Li, Mei Zhang

**Affiliations:** ^1^ State Key Laboratory For Innovation and Transformation of Luobing Theory Key Laboratory of Cardiovascular Remodeling and Function Research of MOE NHC CAMS and Shandong Province Department of Cardiology Qilu Hospital of Shandong University Jinan China; ^2^ Department of Geriatric Medicine, The Second Hospital Cheeloo College of Medicine Shandong University Jinan China; ^3^ Department of Cardiology, The Second Hospital Cheeloo College of Medicine Shandong University Jinan China; ^4^ Human Phenome Institute Zhangjiang Fudan International Innovation Center Fudan University Shanghai China; ^5^ Clinical Epidemiology Unit Qilu Hospital of Shandong University Jinan China; ^6^ Department of Medical Oncology Qilu Hospital of Shandong University Jinan China

**Keywords:** breast cancer, cancer therapy‐related cardiac dysfunction, Crocus total glucosides tablets, left ventricular global longitudinal strain, left ventricular ejection fraction

## Abstract

Crocin shows cardioprotective potential against chemotherapy‐related cardiotoxicity, but clinical evidence for cycle‐synchronized intervention remains limited. This randomized, double‐blind, placebo‐controlled trial investigated whether crocus total glucosides tablets (CTGT) administered synchronously with chemotherapy could prevent cancer therapy‐related cardiac dysfunction (CTRCD) in breast cancer patients. A total of 120 patients were randomly assigned (1:1) to receive either CTGT or placebo for 8 days/cycle, starting 1 day before chemotherapy. Primary endpoints were 6‐month relative decline in left ventricular global longitudinal strain (ΔLVGLS%) and change in left ventricular ejection fraction (ΔLVEF); secondary endpoints included 3‐month measures, LVEF and LVGLS, CTRCD incidence [LVEF<50% or ΔLVGLS%>15% or elevated high‐sensitivity cardiac troponin I (hs‐cTnI)/N‐terminal pro‐B‐type natriuretic peptide (NT‐proBNP)], and severe arrhythmias. Based on the intention‐to‐treat analysis, CTGT significantly attenuated the 6‐month ΔLVGLS% compared with placebo (−6.55 ± 10.85 vs. −12.63 ± 13.49%, *p *= 0.009), whereas ΔLVEF did not differ significantly (−3.67 ± 5.75 vs. −2.40 ± 5.47%, *p *= 0.220). Similar results were observed at 3 months (ΔLVGLS%: −3.91 ± 10.18 vs. −7.76 ± 10.64%, *p *= 0.047). CTGT also reduced the proportion of patients with ΔLVGLS%>15% (18.3 vs. 40.0%, *p *= 0.009) and the incidence of CTRCD (21.7 vs 46.7%, *p *= 0.004). An 8‐day, cycle‐synchronized CTGT regimen attenuates LVGLS decline and reduces CTRCD incidence, highlighting its cardioprotective effect during breast cancer chemotherapy (NCT05504148).

## Graphical abstract

*Cycle‐synchronized CTGT therapy attenuates LVGLS decline and reduces CTRCD in breast cancer patients*. This randomized, double‐blind, placebo‐controlled trial assessed whether cycle‐synchronized CTGT prevent CTRCD in 120 breast cancer patients receiving chemotherapy. Patients received CTGT or placebo for 8 days per cycle, starting 1 day before chemotherapy. Cardiac function was assessed at baseline, 3 months, and 6 months using echocardiography, electrogram, and laboratory tests. The primary endpoints were ΔLVEF and ΔLVGLS% at 6 months. At 3 and 6 months, CTGT significantly attenuated ΔLVGLS% and reduced CTRCD incidence (21.7 vs. 46.7%, *p* = 0.004) compared with placebo. CTRCD‐free survival was also improved. These findings support the use of short‐term, cycle‐synchronized CTGT as a cardioprotective strategy during breast cancer chemotherapy. CTGT, crocus total glucosides tablets; CTRCD, cancer therapy‐related cardiac dysfunction; LVEF, left ventricular ejection fraction; LVGLS, left ventricular global longitudinal strain.

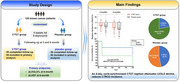

## 1 Introduction

Advances in early detection and treatment have significantly improved breast cancer (BC) outcomes, with 5‐year survival rates now exceeding 90%. Consequently, a growing population of BC survivors faces long‐term health risks, particularly cardiovascular disease, the leading cause of death beyond 10 years postdiagnosis [1]. A meta‐analysis of 142 studies confirmed that BC survivors have a significantly higher risk of cardiovascular death within the first 5 years compared with cancer‐free controls [2]. An important contributor is cancer therapy‐related cardiac dysfunction (CTRCD), commonly associated with chemotherapies such as anthracyclines and human epidermal growth factor receptor 2 (HER2) inhibitor. Anthracycline‐induced cardiotoxicity may present acutely as an asymptomatic decline in left ventricular ejection fraction (LVEF) or as late‐onset cardiomyopathy years after treatment [3]. A recent longitudinal study of 829 BC survivors reported that the prevalence of cardiac dysfunction (LVEF <50%) rose steadily from 1.8% at 2 years to 15.3% at 15 years posttreatment [4]. Combination therapies further elevate this risk. A population‐based study of older women found adjusted 3‐year incidence rates of heart failure or cardiomyopathy of 32.1 per 100 patients for trastuzumab alone and 41.9 per 100 for anthracycline plus trastuzumab, underscoring the urgent need for early identification and management of CTRCD [5].

Conventional surveillance relies mainly on serial LVEF monitoring, but LVEF has limited sensitivity for detecting subclinical myocardial injury, often identifying dysfunction only after significant functional decline. In contrast, left ventricular global longitudinal strain (LVGLS) has emerged as a more sensitive and reproducible marker of early myocardial dysfunction. In the SUCCOUR trial, a LVGLS‐guided strategy initiating cardioprotective therapy at a relative LVGLS reduction ≥12% significantly reduced CTRCD incidence and better preserved LVEF compared with an LVEF‐guided approach [6]. Similarly, the SUCOUR‐MRI trial showed that initiating therapy in patients with isolated LVGLS impairment and preserved LVEF led to better LVEF preservation at 12 months [7]. Current guidelines now endorse LVGLS for early detection of CTRCD, given its greater sensitivity. The 2022 European Society of Cardiology (ESC) cardio‐oncology guidelines classify isolated LVGLS impairment with preserved LVEF as mild CTRCD and give a Class IIa recommendation for considering cardioprotective therapy in such patients [8].

Despite monitoring advances, optimal strategies for preventing CTRCD remain uncertain [9]. Dexrazoxane is recommended for high‐risk patients or those requiring high cumulative anthracycline doses. However, its routine use remains limited due to concerns about antitumor efficacy interference and potential safety risks [10]. Studies have investigated antifibrotic approaches and cardiovascular risk management using β‐blockers, angiotensin‐converting enzyme inhibitors or angiotensin receptor blockers (ACEI/ARB), and statins during BC therapy, but evidence remains inconsistent. For example, enalapril did not significantly prevent myocardial injury or LVEF decline in the PROACT trial [11]. Carvedilol showed mixed results, reducing troponin and diastolic dysfunction in some studies but failing to prevent LVEF decline in others such as the CECCY trial [12]. The Cardiac CARE trial found that combination therapy with ACEI/ARB and β‐blockers did not significantly improve LVEF preservation over standard care alone [13]. Statins have also yielded conflicting data, with atorvastatin reducing cardiotoxicity risk in STOP‐CA but showing no benefit in SPARE‐HF [14, 15]. Emerging agents such as sacubitril/valsartan and sodium‐glucose cotransporter 2 inhibitors show preclinical and early clinical promise, but large‐scale randomized trials are still ongoing [16, 17]. Overall, no universally effective preventive strategy has been established, highlighting a significant gap in reliable CTRCD prevention and underscoring the need for novel evidence‐based interventions.

Among natural compounds studied for cardio‐protection, *Crocus sativus* L. (saffron) and its main bioactive component, crocin, have attracted attention for their antioxidant and anti‐inflammatory properties [18]. Crocus total glucosides tablets (CTGT), a standardized saffron extract, is an approved botanical drug in China for cardiovascular diseases, including angina in chemotherapy patients. In rat models, crocin alleviated doxorubicin‐induced cardiotoxicity by decreasing oxidative stress, enhancing antioxidant defenses, and inhibiting inflammatory factors, thereby preserving myocardial structure and function, as reflected by improved LVEF, lower cardiac enzyme release, and reduced histopathological damage [19, 20, 21]. These results suggest crocin may help prevent CTRCD. In a recent clinical trial, Li et al. reported that 6 months of crocin supplementation reduced the incidence of LVEF decline (≥10%) in BC patients receiving anthracycline‐based chemotherapy [22]. While these results are encouraging, the study did not report rates of LVEF <50% or changes in LVGLS which has been recommended for early detection of asymptomatic CTRCD. Therefore, further research is needed to more fully evaluate the potential role of crocin in preventing CTRCD in this population.

Chemotherapy‐induced cardiomyocyte damage exhibits an early window of injury initiation. For anthracyclines, key mechanisms including topoisomerase IIβ inhibition and oxidative stress are rapidly triggered upon infusion, leading to acute cardiac injury typically within 2–3 days [23]. This provides a rationale for the synchronous administration of cardioprotective agents during chemotherapy to enable real‐time intervention. Given that crocin is a valuable herbal medicine and continuous daily prophylaxis would be costly, we designed a pragmatic cycle‐synchronized regimen (8 days/cycle) to achieve cardioprotection while minimizing financial burden. We therefore conducted a randomized trial to evaluate this abbreviated regimen.

## 2 Results

### 2.1 Study Participants and Follow‐Up

**FIGURE 1:**
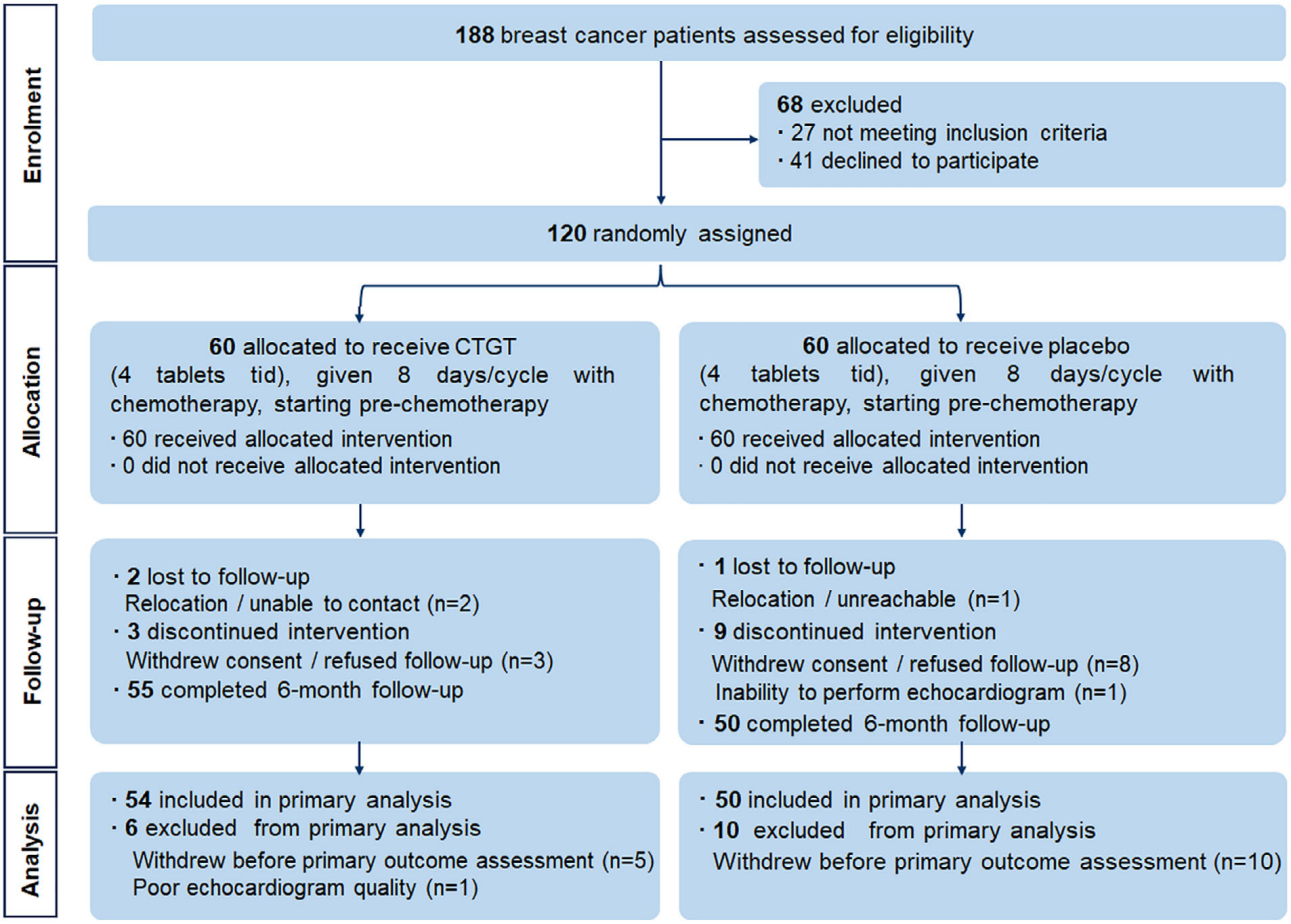
Flow chart diagram of patient screening and follow‐up. All the patients who underwent randomization were included in the primary intention‐to‐treat analysis. CTGT, crocus total glucosides tablets.

From March 29, 2021 to April 26, 2023, a total of 120 patients were enrolled in the study and randomly assigned to receive either CTGT (*n* = 60) or placebo (*n* = 60) (Figure 1). All randomized participants were included in the primary intention‐to‐treat (ITT) analysis for all efficacy and safety outcomes. The mean age of the study population was 48.97 ± 1.00 years. Baseline characteristics included a history of hypertension in 12 patients (10.0%), diabetes in nine (7.5%), coronary artery disease in five (4.2%). Prior treatment with ACE inhibitors/ARBs or β‐receptor blockers in 2 patients each (1.7%). No patients had a history of heart failure. All participants had a confirmed diagnosis of invasive ductal carcinoma of the breast, and 52 (43.3%) exhibited HER2‐positive status.

The two groups were well balanced at baseline, with no statistically significant differences in demographic characteristics, comorbidities, BC pathology (type and stage), chemotherapy regimens, or clinical parameters (echocardiographic and laboratory findings) (all *p* > 0.05; Table 1). During the 6‐month follow‐up period, no participant in either group initiated therapy with ACEIs/ARBs, ß‐blockers, or statins, and dosages remained unchanged for the small subset of patients already receiving these medications at baseline. In addition, no statistically significant difference was observed in the equivalent cumulative dose of anthracyclines between the two groups [CTGT group: 324.08 (300.12, 348.05) mg/m^2^ vs. placebo group: 312.36 (297.49, 327.24) mg/m^2^, *p* = 0.059].

**TABLE 1:** Clinical characteristics of the patients at baseline.

Parameters	CTGT group (*n* = 60)	Placebo group (*n* = 60)
Age(years)	48.75 ± 1.39	49.33 ± 1.52
Heart rate (bpm)	76.33 ± 1.27	76.02 ± 1.51
SBP (mmHg)	120.00 (116.00–121.00)	120.00 (116.00–122.00)
DBP (mmHg)	75.00 (70.00–80.00)	76.00 (73.00–80.00)
BMI (kg/m^2^)	25.39 (21.46–28.12)	23.62 (22.29–26.04)
BSA (m^2^)	1.68 ± 0.03	1.68 ± 0.02
**Comorbidity (*n*, %)**		
Hypertension	8 (13.3%)	4 (6.7%)
Diabetes	2 (3.3%)	7 (11.7%)
Coronary artery disease	1 (1.7%)	4 (6.7%)
**Medication history (*n*, %)**		
ACEI/ARB	1 (1.7%)	1 (1.7%)
β‐RB	1 (1.7%)	1 (1.7%)
CCB	5 (8.3%)	3 (5.0%)
Statins	0 (0)	1 (1.7%)
Aspirin	0 (0)	1 (1.7%)
**Breast cancer pathology**		
Tumor site: Left (*n*, %)	29 (48.3%)	30 (50.0%)
Pathological stage (I/II/III/IV) (*n*, %)	9/31/16/4 (15.0%/51.7%/26.7%/6.7%)	10/33/15/2 (16.7%/55.0%/25.0%/3.3%)
HER2+ (*n*, %)	27 (45.0%)	25 (41.7%)
Ki‐67 labeling index	40% (20%, 60%)	40% (20%, 60%)
**Chemotherapy regimen (*n*, %)**		
A(E)C‐PH/TH ± pertuzumab	10 (16.7%)	11 (18.3%)
TCbH ± pertuzumab	15 (25.0%)	9 (15.0%)
A(E)C‐P/T	35 (58.3%)	38 (63.3%)
TC	0 (0%)	2 (3.3%)
Cumulative dose of anthracyclines (mg/m^2^)^a^	324.08 (300.12, 348.05)	312.36 (297.49, 327.24)
**Echocardiogram**		
IVST (mm)	9.36 ± 1.37	9.35 ± 1.44
PWT (mm)	8.40 ± 1.53	8.77 ± 1.95
LVEDV (mL)	65.83 ± 14.80	67.15 ± 11.99
LVESV (mL)	23.04 ± 6.77	24.17 ± 4.91
LVEF (%)	65.16 ± 4.72	63.87 ± 4.55
LVGLS (%)	−20.77 ± 2.37	−21.28 ± 3.21
*E* (cm/s)	74.12 ± 16.11	77.25 ± 15.86
*A* (cm/s)	75.05 ± 19.05	72.97 ± 16.30
Septal‐e′ (cm/s)	8.64 ± 2.61	9.19 ± 2.51
Lateral‐e′ (cm/s)	12.49 ± 3.43	12.80 ± 3.65
*E*/*A*	1.04 ± 0.31	1.12 ± 0.38
*E*/*e*′	7.47 ± 3.45	7.37 ± 2.07
LAVI (mL/m^2^)	16.35 ± 5.13	16.80 ± 5.81
TAPSE (mm)	20.87 ± 4.76	22.09 ± 3.37
RVFAC (%)	45.74 ± 14.52	48.85 ± 11.54
**Laboratory test**		
WBC (×10^9^/L)	6.13 ± 0.30	6.13 ± 0.18
Neutrophil ratio (%)	64.03 ± 1.23	61.86 ± 1.09
Platelets (×10^9^/L)	272.54 ± 8.36	289.77 ± 9.39
Hemoglobin (g/L)	130.00 (121.00–140.50)	131.00 (124.50–137.00)
ALT (U/L)	15.00 (10.00–20.00)	15.00 (11.50–24.00)
AST (U/L)	16.00 (13.00–21.50)	18.00 (15.00–21.00)
Albumin (g/L)	46.10 (44.10–48.35)	47.50 (45.95–48.40)
Fasting blood glucose (mmol/L)	5.22 (4.79–5.60)	5.00 (4.80–5.34)
Creatinine (µmol/L)	55.05 ± 1.25	54.28 ± 1.16
CCr (mL/min)	112.73 ± 3.54	112.19 ± 4.83
Total cholesterol (mmol/L)	5.17 ± 0.14	5.21 ± 0.17
Triglyceride (mmol/L)	1.09 (0.75–1.49)	1.17 (0.84–1.67)
LDL‐C (mmol/L)	3.03 ± 0.11	3.05 ± 0.11
HDL‐C (mmol/L)	1.46 ± 0.39	1.43 ± 0.05
cTnI (ng/L)	1.29 (0.23–1.91)	1.42 (0.69–2.01)
NT‐proBNP (ng/mL)	44.70 (29.85–80.84)	49.04 (26.30–64.11)
CK (U/L)	59.00 (44.50–73.50)	59.00 (46.50–74.00)
CK‐MB (ng/mL)	0.80 (0.60–1.60)	0.80 (0.55–1.16)
K^+^ (mmol/L)	4.25 ± 0.04	4.30 ± 0.04
Na^+^ (mmol/L)	141.00 (140.00–141.50)	141.00 (140.00–142.00)
D‐Dimer (µg/mL)	0.33 (0.16–0.92)	0.26 (0.16–0.59)
PT‐INR	0.94 (0.92–0.98)	0.93 (0.90–0.97)
APTT (s)	32.51 ± 0.43	31.60 ± 0.39

Continuous variables conforming to normal distribution were expressed as mean ± SD when conforming to normal distribution, otherwise expressed as the median (interquartile range) [*M*(*Q*1–*Q*3)].

*Abbreviations*: *A*, peak late diastolic flow velocity in the mitral inflow spectrum; ACEI, angiotensin converting enzyme inhibitor; ALT, alanine aminotransferase; APTT, activated partial thromboplastin time; ARB, angiotensin receptor blocker; AST, aspartate aminotransferase; β‐RB, β‐receptor blocker; BMI, body mass index; BSA, body surface area; CAD, coronary artery disease; CCB, calcium channel blocker; Ccr, creatinine clearance rate; CK, creatine kinase; CK‐MB, creatine kinase‐MB; CTGT, crocus total glucosides tablets; cTnI, cardiac troponin I; DBP, diastolic blood pressure; E, peak early diastolic flow velocity in the mitral valve spectrum; *e*′, early diastolic myocardial velocities at the septal and lateral mitral annulus by tissue Doppler; HDL‐C, high‐density lipoprotein cholesterol; IVST, interventricular septum thickness; LAVI, left atrial volume index; LDL‐C, low‐density lipoprotein cholesterol; LVEDV, left ventricular end‐diastolic volume; LVEF, left ventricular ejection fraction; LVESV, left ventricular end‐systolic volume; LVGLS, left ventricular global longitudinal strain; NT‐proBNP, N‐terminal pro‐B type natriuretic peptide; PT‐INR, international normalization ratio of prothrombin time; PWT, left ventricular posterior wall thickness; RVFAC, right ventricular fractional area change; SBP, systolic blood pressure; TAPSE, tricuspid annular plane systolic excursion; WBC, white blood cells. The chemotherapy regimen acronym stands for the following medications: A(E)C‐PH/TH: A, doxorubicin; E, epirubicin; C, cyclophosphamide; P, paclitaxel; T, docetaxel; H, trastuzumab; TCbH: T, docetaxel; Cb, carboplatin; H, trastuzumab; TC: T, docetaxel; C (Cb), carboplatin.

^a^The cumulative dose of anthracyclines was analyzed in 120 patients who completed the trial. Among them, 49 patients from the placebo group and 45 from the CTGT group, all of whom received the anthracycline‐based regimen, were ultimately included in the calculation.

During the double‐blind treatment interval, study drug discontinuation or exclusion from the per‐protocol analysis occurred in 16 participants (13.3%): 6 () (10.0%) in the CTGT group and 10 (16.7%) in the placebo group (*p* = 0.28). Reasons for discontinuation included withdrawal of consent (CTGT, *n* = 3; placebo, *n* = 7), loss to follow‐up (CTGT, *n* = 2; placebo, *n* = 1), and inability to undergo echocardiography due to radiation‐induced skin damage (placebo, *n* = 1). Notably, no discontinuations resulted from cardiovascular deterioration or treatment‐related adverse events. Additionally, one CTGT participant completed all assessments but was excluded posthoc due to poor data quality secondary to multiple comorbidities (classified as an exclusion rather than a discontinuation in Figure 1). Detailed reasons for individual‐level discontinuation are listed in Tables S4.1 and S4.2. A total of 104 participants, including 54 in the CTGT group and 50 in the placebo group, completed the 6‐month follow‐up and constituted the per‐protocol population, the analysis of which is presented in the Supplementary Material 5.

### 2.2 Primary Outcomes

**FIGURE 2:**
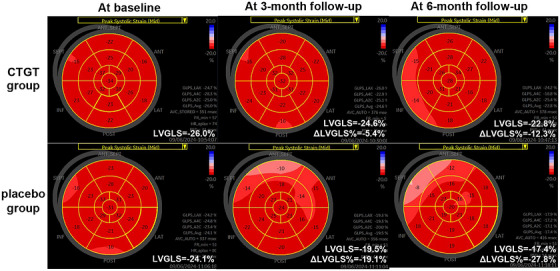
Representative echocardiographic images of LVGLS of patients in the CTGT group and placebo group. A 52‐year‐old patient in the CTGT group (upper row) showed a relative decline of LVGLS from baseline by −5.4% at 3‐month follow‐up and −12.3% at 6‐month follow‐up, respectively. A 52‐year‐old patient in the placebo group (lower row) showed a relative decline of LVGLS from baseline by −19.1% at 3‐month follow‐up and −27.8% at 6‐month follow‐up respectively. The chemotherapy regimen for both patients was A (E) C sequential PH/TH regimen. CTGT, crocus total glucosides tablets; LVGLS, left ventricular global longitudinal strain.

At the 6‐month follow‐up, the relative decline in LVGLS from baseline was significantly attenuated in the CTGT group compared with the placebo group [estimated marginal mean (EMM) ΔLVGLS%: −6.55% (95% CI: −9.42 to −3.68) vs. −12.63% (95% CI: −15.47 to −9.79), *p* = 0.009] (Figure 2). This difference remained significant after Hochberg adjustment for multiple comparisons. Conversely, the coprimary endpoint—the reduction in LVEF percentage points, did not differ significantly between groups [EMM ΔLVEF: −3.67% (95% CI: −5.16 to −2.18) vs. −2.40% (95% CI: −3.91 to −0.89), *p* = 0.220] (Table 2).

**TABLE 2:** Primary and secondary cardiovascular outcomes.

Parameters	CTGT group (*n* = 60)	Placebo group (*n* = 60)	*p* Value
**Primary outcome event**			
Reduced percentage points of LVEF at 6 months (%)	−3.67 ± 5.75	−2.40 ± 5.47	0.220
Relative decline in LVGLS at 6 months (%)	−6.55 ± 10.85	−12.63 ± 13.49	0.009
**Secondary outcome event**			
Reduced percentage points of LVEF at 3 months (%)	−2.17 ± 4.05	−1.21 ± 3.86	0.189
Relative decline in LVGLS at 3 months (%)	−3.91 ± 10.18	−7.76 ± 10.64	0.047
LVEF at 3 months (%)	63.53 ± 3.81	63.02 ± 4.64	0.518
LVGLS at 3 months (%)	−15.86 ± 2.34	−19.59 ± 3.35	0.611
LVEF at 6 months (%)	62.03 ± 5.29	61.83 ± 5.14	0.839
LVGLS at 6 months (%)	−19.30 ± 2.36	−18.51 ± 3.36	0.142
LVEF decline to <50% (*n*, %)	0(0)	0(0)	
New relative decline in LVGLS by >15% from baseline (*n*, %)	11(18.3%)	24(40.0%)	0.009
hs‐cTnI > ULN (*n*, %)	3(3.7%)	4(8.0%)	0.999
NT‐proBNP > ULN of the corresponding age group (*n*, %)	0(0)	0(0)	—
Newly emerging severe arrhythmias (*n*, %)	0(0)	0(0)	—
Incidence of CTRCD	13(21.7%)	28 (46.7%)	0.004

Continuous variables conforming to normal distribution were expressed as mean ± SD when conforming to normal distribution, otherwise expressed as the median (interquartile range) [*M*(*Q*1, *Q*3)].

Abbreviations: cTnI, cardiac troponin I; CTGT, crocus total glucosides tablets; CTRCD, cancer therapy‐related cardiac dysfunction; LVEF, left ventricular ejection fraction; LVGLS, left ventricular global longitudinal strain; NT‐proBNP, N‐terminal pro‐B type natriuretic peptide; ULN, upper limit of normal.

### 2.3 Secondary Outcomes

At the 3‐month follow‐up, the relative decline in LVGLS from baseline was significantly attenuated in the CTGT group compared with the placebo group [EMM ΔLVGLS%: −3.91% (95% CI: −6.61 to −1.21) vs. −7.76% (95% CI: −10.43 to −5.09), *p* = 0.047]. In contrast, the reduction in LVEF percentage points did not differ significantly between groups [EMM ΔLVEF: −2.17% (95% CI: −3.24 to −1.10) vs. −1.21% (95% CI: −2.29 to −0.13), *p* = 0.189] (Table 2).

At both 3‐ and 6‐month follow‐ups, LVGLS values were all significantly better preserved in the CTGT group than in the placebo group (*p* = 0.039 and *p* = 0.033, respectively; Table 2) (Figure 2). A repeated measures analysis of variance (RM‐ANOVA) assessing LVGLS over time revealed a significant group‐by‐time interaction (*p* for interaction = 0.019; Table S6.1), indicating that LVGLS trajectories differed between groups across time. In contrast, no significant between‐group differences were observed for LVEF at either time point (Table 2), and the group‐by‐time interaction for LVEF was not significant (*p* for interaction = 0.375; Table S6.2).

**FIGURE 3:**
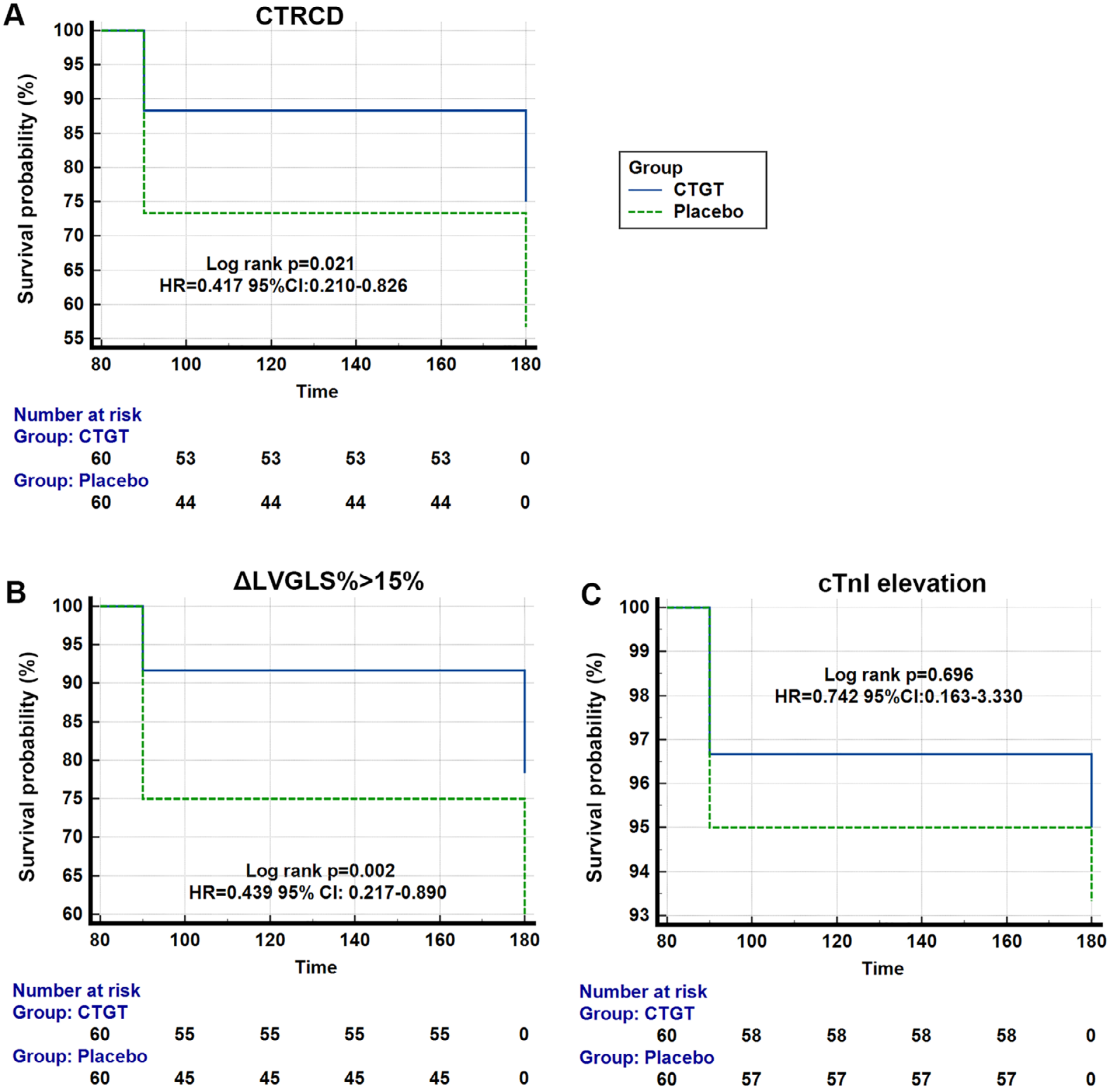
Kaplan–Meier survival curves for CTRCD, ΔLVGLS%, and cTnI elevation in the placebo group and CTGT group. Kaplan–Meier curves displayed the cumulative survival probability (%) for (A) CTRCD, (B) the absolute ΔLVGLS% >15%, and (C) cTnI elevation in the placebo group (dashed green line, *n* = 60) and the CTGT group (solid blue line, *n* = 60) over time. The number of patients at risk at each time point is shown below each panel. Hazard ratios (HR) with 95% confidence intervals (CI) were estimated using a Cox proportional hazards model.  CTGT, crocus total glucosides tablets; cTnI, cardiac troponin I; CTRCD, cancer therapy‐related cardiac dysfunction; ΔLVGLS%, the relative decline in left ventricular global longitudinal strain from baseline.

During the 6‐month follow‐up, the occurrence of a new relative decline in LVGLS exceeding 15% was significantly lower in the CTGT group compared with the placebo group [11/60 (18.3%) vs. 24/60 (40.0%), *p *= 0.009]. No participants in either group developed LVEF decline to <50% (Table 2). New hs‐cTnI elevations (>ULN) were observed in three patients (3.7%) in the CTGT group and four (8.0%) in the placebo group (*p *= 0.999). Neither group exhibited new NT‐proBNP elevations above the ULN of the corresponding age nor new‐onset severe arrhythmias. Consequently, the incidence of CTRCD was significantly lower in the CTGT group compared with the placebo group (21.7 vs. 46.7%, *p *= 0.004). Kaplan–Meier analysis demonstrated a significantly higher CTRCD‐free survival probability in the CTGT group compared with the placebo group [HR = 0.417, 95% CI: 0.210–0.826; log‐rank *p* = 0.021] (Figure 3A). Significant differences in survival probability were also observed for ΔLVGLS% >15% [HR = 0.439, 95% CI: 0.217–0.890, log‐rank *p* = 0.002] (Figure 3B), whereas there was no significant difference in survival probability for cTnI elevation between the two groups [HR = 0.742, 95% CI: 0.163–3.330, log‐rank *p* = 0.696] (Figure 3C).

### 2.4 Subgroup Analyses by Chemotherapy Regimen and Comorbidities

**FIGURE 4:**
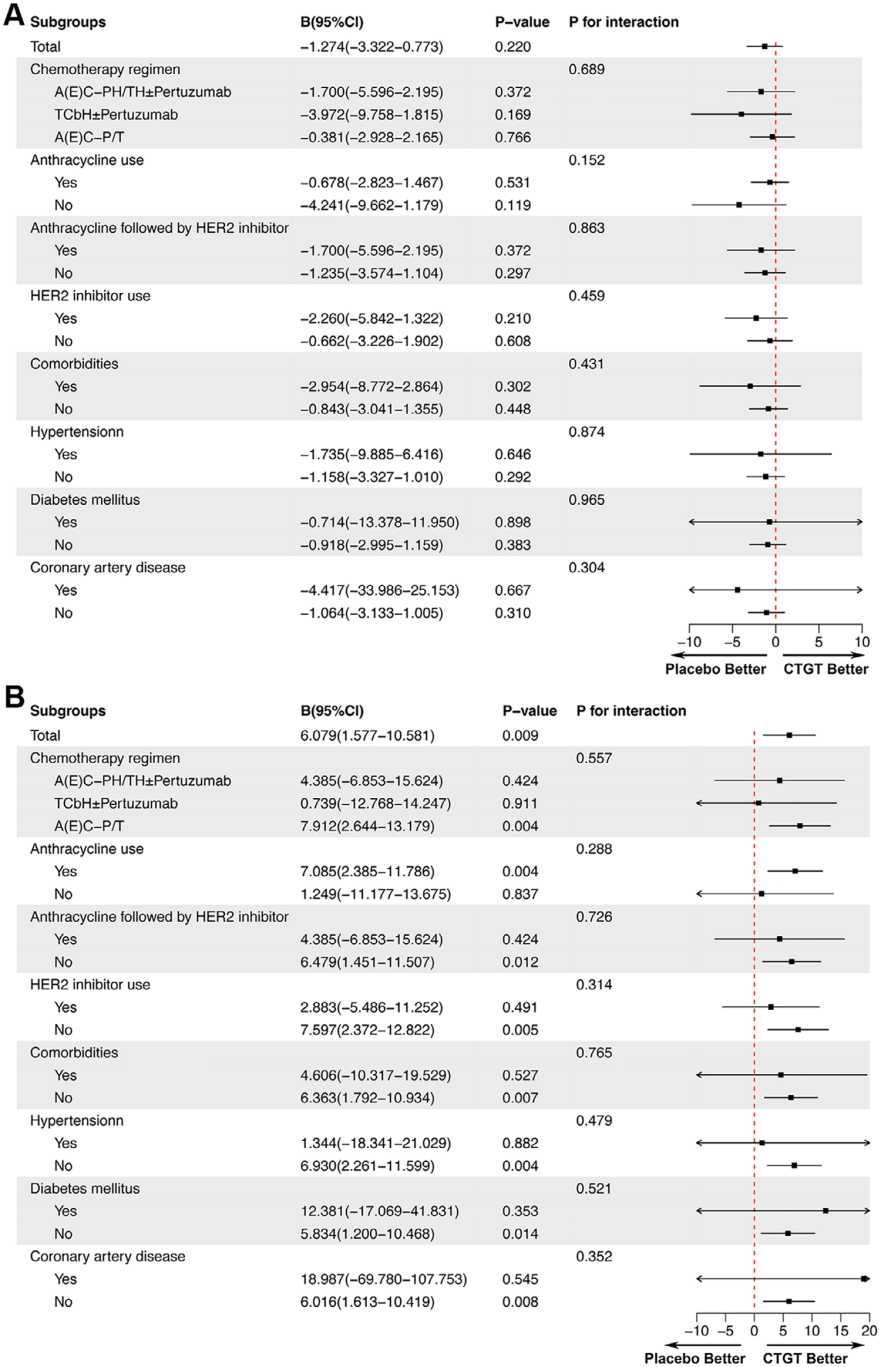
Subgroup analysis of the treatment effect on ΔLVEF and ΔLVGLS% at 6 months. Forest plot showing the estimated treatment effect (*B* value with 95% confidence interval) of CTGT versus placebo on the primary endpoint, ΔLVEF (A) and ΔLVGLS% (B) at 6 months, across predefined subgroups. *p* Values for interaction test whether the treatment effect differs significantly between subgroups. CTGT, crocus total glucosides tablets; HER2, human epidermal growth factor receptor 2; ΔLVEF, change in left ventricular ejection fraction; ΔLVGLS%, relative decline in left ventricular global longitudinal strain.

To evaluate potential heterogeneity in the cardioprotective effect of CTGT, predefined subgroup analyses were performed based on chemotherapy type, anthracycline use, and HER2 inhibitor use (Figure 4). The protective effect of CTGT on ΔLVGLS% at 6 months was consistently observed across most chemotherapy subgroups, with no significant treatment‐by‐subgroup interactions detected for any chemotherapy‐related factor (all *p* for interaction > 0.05), indicating a generally homogenous effect. However, when examining specific subgroups, the estimated treatment differences (*B* values) for ΔLVGLS% significantly favored CTGT in patients receiving anthracycline‐based regimens [*B* = 7.085 (95% CI: 2.385–11.786), *p *= 0.004] and in those not receiving HER2 inhibitors [*B* = 7.597 (95% CI: 2.372–12.822), *p *= 0.005]. Conversely, no significant between‐group differences in ΔLVEF at 6 months were observed across any chemotherapy‐related subgroups, and no significant treatment‐by‐subgroup interactions were detected (*p* for interaction > 0.05 for all).

Similarly, subgroup analyses based on key comorbidities (hypertension, diabetes mellitus, and coronary artery disease) revealed a consistent cardioprotective effect of CTGT on ΔLVGLS% at 6 months, without any significant treatment‐by‐subgroup interactions (all *p* for interaction > 0.05). This suggests that the benefit of CTGT on LVGLS is largely independent of these common comorbidities. Regarding ΔLVEF, no significant differences between the CTGT and placebo groups were observed in any comorbidity subgroups at 6 months, and no significant treatment‐by‐subgroup interactions were identified (*p* for interaction > 0.05 for all).

### 2.5 Adverse Events

The incidence of adverse events did not differ significantly between the two groups. According to the Common Terminology Criteria for Adverse Events (CTCAE) version 5.0 [24], the severity of leukopenia and anemia, which were numerically higher in the CTGT group, was predominantly Grade 1 or 2, with no significant between‐group differences in the incidence of Grade ≥ 3 events (Table 3). No participants discontinued treatment due to drug‐related adverse effects.

**TABLE 3:** Adverse events.

Adverse events (*n*, %)	CTGT group (*n* = 60)	Placebo group (*n* = 60)	*p* Value
Gastrointestinal reaction	11 (18.3%)	10 (16.7%)	0.810
Rash	1 (1.7%)	0 (0)	1.000
Fatigue	16 (26.7%)	16 (26.7%)	1.000
Hepatic injury	1 (1.7%)	3 (5.0%)	0.309
Kidney injury	0 (0)	0 (0)	—
**Leukocytopenia**	16 (26.7%)	9 (15.0%)	0.116
Grade 1	5(8.3%)	3(5.0%)	0.439
Grade 2	10(16.7%)	5(8.3%)	0.168
Grade 3	1(1.7%)	1(1.7%)	0.999
Grade 4	0	0	
Grade 5	0	0	
**Anemia**	20 (33.3%)	12 (20.0%)	0.099
Grade 1	14(23.3%)	10(16.7%)	0.361
Grade 2	6(10.0%)	1(1.7%)	0.114
Grade 3	0	1(1.7%)	0.999
Grade 4	0	0	
Grade 5	0	0	
Major bleeding	0 (0.0%)	0 (0.0%)	—
Emerging activity‐related chest pain and chest tightness	2 (3.3%)	2 (3.3%)	1.000
Withdrew due to study drugs	0 (0.0%)	0 (0.0%)	—

Adverse events were graded according to the Common Terminology Criteria for Adverse Events (CTCAE) version 5.0 [24].

*Abbreviation*: CTGT, crocus total glucosides tablets.

### 2.6 Inter‐observer and Intraobserver Variability

Excellent reproducibility was demonstrated for both echocardiographic measures. The intraclass correlation coefficients of intraobserver reproducibility for LVEF and LVGLS were 0.802 and 0.920, respectively (Figure S7A,B). The intraclass correlation coefficients for interobserver reproducibility for LVEF and LVGLS were 0.838 and 0.966, respectively (Figure S7C,D).

## 3 Discussion

In this randomized, placebo‐controlled trial, a short 8‐day course of CTGT administered during each chemotherapy cycle provided significant cardiac protection in BC patients. Compared with placebo, the CTGT group exhibited significantly better preservation of LVGLS at 3 and 6 months and a lower incidence of asymptomatic mild CTRCD, primarily defined as a relative decline in LVGLS >15% from baseline. The primary endpoint of ΔLVGLS% remained statistically significant even after applying the Hochberg procedure to control for multiple comparisons, reinforcing the robustness of this finding. These findings highlight the cardioprotective potential of CTGT during the vulnerable window of cancer therapy‐related cardiovascular toxicity (CTR‐CVT). Notably, this brief, cycle‐synchronized intervention achieved these benefits with a favorable safety profile and potential cost‐effectiveness, offering a practical strategy to balance treatment efficacy with cardiovascular safety. As this was an investigator‐initiated trial evaluating a novel, cycle‐synchronized regimen of an already‐marketed botanical drug, these positive results provide a strong rationale for its broader application and for designing larger, definitive studies. Our results suggest that short‐term, chemotherapy‐aligned CTGT administration may serve as an effective and feasible approach to mitigate CTR‐CVT without adding significant treatment burden.

Our study demonstrated that the protective effect of concurrent CTGT against CTRCD is primarily mediated through attenuation of the decline in LVGLS, aligning with the early clinical manifestations of CTRCD in BC patients and the contemporary understanding that subclinical myocardial injury precedes overt systolic dysfunction. LVEF, the traditional surveillance parameter of cardiac dysfunction, demonstrates limited sensitivity for detecting early myocardial injury. In a prospective cardiovascular magnetic resonance (CMR) study of HER2+ BC survivors assessed at a median of 7.8 years post trastuzumab, 25% exhibited LVEF <50% despite all having normal LVEF at treatment completion, confirming that subclinical injury precedes overt functional decline [25]. A large cohort study of BC survivors exposed to cardiotoxic therapy found the cumulative incidence of LVEF‐defined cardiac dysfunction increased slowly from 1.8% at 2 years to 15.3% at 15 years posttreatment [4]. This gradual trajectory suggests that myocardial damage accrues over years while remaining undetected by LVEF alone. Thus, CTRCD in these patients is not nonexistent, but rather undetected in a timely manner. Cardinale et al. assessed LVEF in 2625 patients receiving anthracycline‐containing therapy and reported a 9% overall incidence of cardiotoxicity (LVEF decrease >10 points to <50%) over a median follow‐up of 5.2 years. Following initiation of heart failure therapy, only 11% of affected patients achieved full recovery and 71% partial recovery, highlighting that early detection and prompt treatment are crucial for functional recovery [26]. Consequently, contemporary cardio‐oncology guidelines emphasize the limitations of LVEF for early detection of CTRCD and recommend incorporation of more sensitive markers such as LVGLS and cardiac biomarkers for CTRCD surveillance [8]. LVGLS could detect impairment weeks to months before LVEF declines [27]. Our previous studies found that while LVEF remained >50% through four chemotherapy cycles, LVGLS detected significant functional decline as early as two chemotherapy cycles [28, 29]. A study of BC patients treated with anthracyclines and trastuzumab and followed over 12 months reported that 42% met the prespecified CTRCD criteria based on relative decline of LVGLS >15%, whereas only 22% to 27% met CTRCD criteria based on various LVEF definitions (reduction of ≥10% to <55%, or ≥5% reduction with heart failure symptoms) [30]. Meta‐analyses have demonstrated that a relative LVGLS reduction of 10–15%, or an absolute change of 2–3% from baseline, detects subclinical cardiotoxicity with 80–90% sensitivity and 80% specificity, often before any decline in LVEF is observed [31, 32]. Thavendiranathan et al.’s randomized trial demonstrated superior outcomes with LVGLS‐guided (≥12% relative decline) versus LVEF‐guided (>10% absolute reduction) monitoring in high‐risk anthracycline patients, with the LVGLS group showing higher preventive therapy uptake, lower CTRCD incidence, and attenuated LVEF decline at 1‐year follow‐up, confirming LVGLS's advantage in preventing significant cardiac dysfunction [33]. The SUCCOUR‐MRI trial reinforced this by demonstrating that, in patients with isolated LVGLS reduction after anthracyclines, cardioprotective therapy was associated with better preservation of LVEF compared with usual care [7]. Based on these, the 2022 ESC Guidelines issue a Class IIa recommendation for considering cardioprotective therapy in patients with isolated LVGLS impairment despite preserved LVEF [8]. Consistent with these findings, the protective effect of CTGT against CTRCD in the present trial was manifested as an attenuation of LVGLS decline and a reduction in the incidence of a relative decline in LVGLS >15%, whereas no significant differences in LVEF changes were observed between the two groups. This pattern of results is physiologically plausible: CTGT appears to mitigate the early, subclinical myocardial injury detectable by the sensitive LVGLS marker, an effect that may precede any measurable impact on the less sensitive LVEF. The nonsignificant finding for ΔLVEF, for which the study was formally powered, does not negate the cardioprotective effect but rather highlights the appropriateness of using LVGLS as a more sensitive endpoint in this setting.

In this study, the incidence of cardiac biomarker elevation was relatively low and did not differ significantly between the CTGT and placebo groups. This finding may reflect the distinct patterns of cardiac biomarker release following cardiotoxic chemotherapy. Cardinale et al. found that 30% of patients exhibited troponin I elevation after high‐dose chemotherapy, with 9% showing persistent positivity that predicts an 84% incidence of major cardiac events [34]. The low rate of biomarker‐defined CTRCD in our trial likely reflects this phenomenon, as isolated transient elevations lack specificity for predicting clinically meaningful dysfunction. Furthermore, the diagnostic value of troponin for concurrent CTRCD is limited. Esmaeilzadeh et al. reported that elevations in hs‐cTnI (>26 pg/mL) and B‐type natriuretic peptide (BNP) (≥35 pg/mL) occurred in only 10 and 24% of patients with BC, respectively. Using CMR as the reference standard, hs‐cTnI demonstrated 92% specificity and 24% sensitivity for detecting concurrent CTRCD, while BNP showed similarly high specificity but low sensitivity, indicating that normal values of these biomarkers do not reliably rule out active myocardial injury, nor do minor elevations necessarily signify established cardiac dysfunction [30]. This limited sensitivity for the concurrent diagnosis of CTRCD is consistent with our observation that biomarker‐defined myocardial injury did not differentiate treatment groups, despite clear divergence in GLS‐defined CTRCD. The incidence of troponin elevation in the placebo group was 8.0%, similar to rates previously reported in anthracycline‐treated populations [34]. The numerically lower incidence in the CTGT group (3.7%), although not statistically significant, suggests a possible modest effect on myocardial injury that our study may have been underpowered to detect due to the limited sample size and low event rates. For NT‐proBNP, interpretation is complicated by age‐related reference values. To enhance specificity for clinically meaningful myocardial dysfunction, we therefore applied age‐stratified diagnostic thresholds for heart failure rather than the lower rule‐out cutoff, which may have reduced sensitivity for mild, subclinical elevations but improved specificity for clinically meaningful dysfunction. Together, these findings suggest that while biomarkers provide complementary prognostic information, LVGLS may offer greater sensitivity for detecting both CTRCD and the cardioprotective effects of interventions in clinical trials.

In the present study, enrolled patients received guideline‐endorsed regimens as per the latest European Society for Medical Oncology and American Society of Clinical Oncology recommendations, including anthracycline–taxane sequences and HER2‐directed combinations. The cardiovascular toxicities associated with these therapies are multifaceted and drug specific. Anthracyclines induce dose‐dependent cardiomyocyte injury primarily through topoisomerase 2β inhibition, leading to DNA double‐strand breaks, mitochondrial dysfunction, and the generation of reactive oxygen species [35]. This oxidative stress and inflammatory cascade activation culminate in irreversible cardiomyocyte death, clinically manifesting as left ventricular dysfunction, heart failure, and arrhythmias, with the risk compounded by cyclophosphamide, which can cause myocarditis and atrial fibrillation, particularly at higher doses [8, 36]. HER2‐targeted therapies, notably trastuzumab, bind to HER2 receptors highly expressed on cardiomyocytes, thereby blocking crucial cardioprotective pathways that facilitate survival and stress adaptation [37]. This results in a reversible contractile dysfunction, with the risk of cardiac events escalating significantly when trastuzumab is administered after anthracyclines or in patients with pre‐existing coronary artery disease. Dual HER2 blockade with pertuzumab may further increase this risk. Taxanes (paclitaxel, docetaxel) are associated with sinus bradycardia, atrioventricular block, and, rarely, coronary vasospasm. A common mechanism across these agents is the induction of oxidative stress and inflammation, which collectively potentiate myocardial vulnerability. Despite this mechanistic understanding, effective primary prevention strategies remain limited and contentious. Dexrazoxane, an iron‐chelating agent that inhibits anthracycline‐induced topoisomerase 2β‐mediated DNA damage, is the only approved cardioprotectant. However, its clinical application is severely restricted. As highlighted in the 2022 ESC Cardio‐Oncology Guidelines, its use is typically reserved for patients with advanced BC who have already received a high cumulative anthracycline dose and require further anthracycline‐based therapy, reflecting regulatory restrictions by the European Medicines Agency and the Food and Drug Administration [8]. This limitation stems from enduring concerns regarding dexrazoxane's potential to interfere with antitumor efficacy [38]. The limited use of dexrazoxane reveals the critical absence of a widely accepted and easily applied primary prevention strategy in cardio‐oncology. Moreover, contemporary trials of renin–angiotensin–aldosterone system inhibitors, beta‐blockers, and statins have yielded heterogeneous results, failing to establish a universal prophylactic strategy. These constraints underscore the urgent need for risk‐adapted monitoring and innovative cardio‐oncology rehabilitation models to mitigate cardiovascular toxicity without compromising oncological outcomes in BC patients.

Saffron and its main bioactive component, crocin, have shown cardioprotective potential due to their potent antioxidant and anti‐inflammatory properties [18]. Preclinical evidence has demonstrated that crocin effectively attenuates doxorubicin‐induced myocardial injury by reducing oxidative stress markers such as malondialdehyde, restoring the activity of endogenous antioxidants including superoxide dismutase and catalase, and suppressing proinflammatory mediators like nuclear factor‐kappa B, tumor necrosis factor‐alpha, and interleukin‐1β [39, 40]. A defining feature of crocin is its selective cytoprotective profile, and accumulating evidence indicates that it does not interfere with the antitumor efficacy of doxorubicin. On the contrary, studies suggest that crocin may exert synergistic or additive anticancer effects by enhancing chemosensitivity and inhibiting tumor proliferation, possibly through modulation of redox balance and inflammatory pathways within malignant cells [39, 40]. The dual action of protecting normal tissues while preserving or even potentiating chemotherapeutic activity positions saffron‐derived compounds as uniquely attractive adjuncts in cardio‐oncology. Their safety profile, tolerability, and lack of significant drug–drug interactions further support their translational potential.

Thus, saffron and its constituents represent a novel, mechanism‐based strategy for primary prevention of chemotherapy‐induced cardiovascular toxicity, warranting rigorous clinical evaluation in BC patients receiving anthracycline‐ and HER2‐directed regimens. In this randomized, placebo‐controlled trial, administration of an 8‐day CTGT regimen during each chemotherapy cycle significantly preserved LVGLS and reduced the incidence of asymptomatic CTRCD, with a favorable safety and potential cost‐effectiveness profile. Subgroup analyses revealed no significant interaction between treatment effect and chemotherapy type, suggesting consistent cardioprotective benefits across guideline recommended regimens. However, the small sample sizes in certain subgroups, particularly the TC regimen, limit definitive conclusions regarding its efficacy in these specific settings. These findings support CTGT as a practical and effective primary prevention strategy for CTRCD. Based on current Chinese hospital pricing, the 8‐day per cycle regimen reduces drug cost by approximately 62% per cycle compared with continuous daily prophylaxis (as detailed in Supplementary Material 3.2), offering a clear economic advantage that enhances affordability and patient adherence, and underscore the need for further validation in larger, regimen‐specific cohorts.

Our findings, demonstrating the cardioprotective efficacy of CTGT, align with the evolving paradigm in cardio‐oncology. This field is advancing beyond merely preventing treatment‐related cardiac injury toward a proactive model of “tumor treatment with concurrent cardiac therapy.” The emerging concept of pan‐cardio‐oncology further expands this bidirectional relationship [41]. CTGT, as a safe and effective cardioprotective strategy, exemplifies this shift, warranting further investigation into its potential broader panvascular benefits.

## 4 Limitations

This study has several limitations. First, as a single‐center trial with a relatively small sample size, our findings may be influenced by site‐specific referral patterns, regional patient characteristics, and potential selection bias, which could limit the generalizability of the results. Future multicenter trials should adopt a comprehensive approach to mitigate such biases, including geographically diverse recruitment, standardized imaging protocols with centralized blinded core laboratory reading, and prespecified assessments of treatment‐effect heterogeneity. Second, the sample size was calculated based on the anticipated effect on LVEF, the traditional gold standard. Consequently, the study may have been underpowered to detect statistically significant differences in LVEF. The significant finding for the more sensitive LVGLS endpoint, while robust after multiplicity correction, should ideally be confirmed in a larger trial primarily powered for LVGLS. To address this, we plan to conduct a multicenter, randomized, double‐blind, placebo‐controlled study to further validate the cardioprotective efficacy of CTGT in preventing CTRCD. Third, the current follow‐up period was limited to 6 months after chemotherapy initiation, precluding assessment of long‐term outcomes. We intend to extend follow‐up with annual evaluations in this cohort to examine the potential long‐term cardiovascular benefits of CTGT in BC patients receiving chemotherapy. Fourth, the schedule for measuring hs‐cTnI and NT‐proBNP may not have captured early postinfusion biomarker peaks, and ambulatory electrocardiogram performed only at baseline and 6 months could have missed transient arrhythmias. Consequently, the incidence of acute subclinical myocardial injury may have been underestimated. Future studies with more intensive monitoring, including assessments before and after each chemotherapy cycle, are warranted to better capture these early events.

## 5 Conclusions

An 8‐day/cycle regimen of CTGT administered concurrently with chemotherapy effectively attenuates the relative decline in LVGLS and reduces CTRCD incidence in BC patients. Although the prespecified LVEF endpoint did not differ significantly, the significant preservation of LVGLS, a more sensitive marker of early myocardial dysfunction, supports the cardioprotective potential of this brief, cycle‐synchronized strategy. These findings support its prompt clinical use as a feasible, efficient cardioprotective strategy that reduces CTRCD while minimizing treatment burden and cost.

## 6 Materials and Methods

See Supplementary Materials 1 for details.

### 6.1 | Study Design and Eligibility

This study was a double‐blind, placebo‐controlled randomized clinical trial conducted in Qilu Hospital of Shandong University, China. The study complies with the principles of the Declaration of Helsinki, and was approved by the Research Ethics Committee of Qilu Hospital, Shandong University (ethics approval number: KYLL‐202008‐191) prior to enrollment of the first participant. All participants provided written informed consent. This trial was registered in ClinicalTrials.gov (NCT05504148).

### 6.2 Patients

BC patients who were to receive adjuvant chemotherapy and/or adjuvant targeted therapy at Qilu Hospital of Shandong University were recruited. The inclusion criteria were as follows: (1) 25–80 years old, female; (2) diagnosed as BC by histopathology; (3) intend to receive adjuvant radiotherapy/chemotherapy or combined with adjuvant trastuzumab or pertuzumab; (4) at least six cycles of chemotherapy after enrollment. The exclusion criteria included: (1) pregnant or lactating women; (2) poor image quality of echocardiography that did not allow complete analysis; (3) persistent atrial fibrillation or severe arrhythmia which affect echocardiographic data collection and analysis; (4) participating in other clinical studies of traditional Chinese medicine.

### 6.3 Trial Procedure and Interventions

Eligible patients were randomly assigned in a 1:1 ratio to receive either CTGT or a matching placebo. CTGT is a standardized botanical drug (Reyoung Pharmaceutical Co., Ltd., Zibo, China) containing crocus total glucosides, 12 mg per tablet. The placebo tablets were identical in appearance and manufactured by the same company. In each chemotherapy cycle, patients received either CTGT or placebo (four tablets, three times daily) in a brief 8‐day regimen, beginning 1 day before chemotherapy and continuing for 8 consecutive days. The chemotherapy regimen for each enrolled patient was determined by the oncologist based on the patient's condition and relevant treatment guidelines.

### 6.4 Echocardiography, Electrocardiogram, and Laboratory Assessment

Two‐dimensional echocardiography with speckle tracking was performed at baseline, 3 months, and 6 months. Echocardiograms were acquired by two experienced sonographers using the GE vivid E9 and E95 system (GE Healthcare; Vingmed Ultrasound, Horten, Norway) equipped with an M5S probe (1.5–4.6 MHz), according to the guidelines of the American Society of Echocardiography. Two experienced physicians blinded to group assignment independently analyzed the images using an EchoPAC workstation (Version 204) to measure LVEF and LVGLS. Thirty subjects were randomly selected by two experienced echocardiographers to assess the consistency of the analyzed results. The echocardiographers were blinded to clinical data and to each other's findings. The previously analyzed images were measured again by the same echocardiographer 1 month later to assess intraobserver agreement.

Standard electrocardiograms were obtained at baseline, 3 months, and 6 months, and dynamic electrocardiogram at baseline and 6 months. Laboratory tests including hs‐cTnI, NT‐proBNP, and other routine indices were conducted in the laboratory of Qilu Hospital of Shandong University at baseline, 3 months, and 6 months.

### 6.5 Study Endpoints

The primary endpoints were the relative decline in LVGLS from the baseline (ΔLVGLS%) and absolute reduction in LVEF percentage points (ΔLVEF) at 6 months. The relative decline in LVGLS was calculated as ΔLVGLS% = (|LVGLS_‐follow‐up_| − |LVGLS_‐baseline_|)/|LVGLS_‐baseline_| × 100%. The absolute LVEF reduction was calculated as ΔLVEF = LVEF_‐follow‐up_ − LVEF_‐baseline_.

Secondary endpoints included: (1) ΔLVGLS% and ΔLVEF at 3 months; (2) LVEF and LVGLS values at both 3‐month and 6‐month follow‐ups; (3) the incidence of CTRCD, defined as either a reduction in LVEF to <50% or a new relative decline in LVGLS by >15% from baseline using absolute values [8], along with the incidence of newly elevated hs‐cTnI above the upper limit of normal (ULN) and/or NT‐proBNP exceeding the age‐specific ULN; and (4) the occurrence of newly emerging severe arrhythmias during the 6‐month follow‐up period.

Security endpoints included any or more of the following new exceptions: ALT ≥ 3 ULN, serum creatinine level increased by ≥30%, digestive tract symptoms such as nausea, vomiting and diarrhea, hemoglobin level ≤90 g/L, severe bleeding (Bleeding Academic Research Consortium Grade 3 or above), activity‐related chest pain and chest tightness. Adverse events were graded according to the CTCAE version 5.0. [24]

### 6.6 Sample Size Analysis

Sample size was calculated using PASS 11.0.7 with a two sided α of 0.05, 80% power (*β* = 0.2), and a 1:1 allocation ratio. Based on preliminary data showing a mean ΔLVEF of 3.28% (SD 5.38%) in untreated controls versus an anticipated ΔLVEF of ≤0.17% in the CTGT group at 6 months, we estimated a required sample size of 48 pairs. To account for a 20% dropout rate, 120 patients were ultimately enrolled.

### 6.7 Statistical Analysis

Data were analyzed using SPSS version 25.0 (IBM, Armonk, New York) and R version 4.4.1(R Foundation for Statistical Computing, Vienna, Austria). Normally distributed continuous variables were expressed as mean ± SD and compared using independent samples *t*‐tests; non‐normally distributed variables were expressed as median (interquartile range) [*M*(*Q*1, *Q*3)]. Categorical variables were expressed as percentages and analyzed using the Chi‐square test. The primary and secondary efficacy analyses followed the ITT principle. Missing data were handled by multiple imputation with chained equations under the missing at random assumption, generating 50 imputed datasets. Results were pooled using Rubin's rules. For longitudinal trajectories of LVEF and LVGLS at baseline, 3 months, and 6 months, repeated measures ANOVA with Bonferroni correction was used. To control the family wise error rate for the two coprimary endpoints, a Hochberg procedure was applied. Secondary endpoints were analyzed without multiplicity adjustment. For time‐to‐event outcomes (CTRCD, ΔLVGLS% >15%, hs‐cTnI elevation), Kaplan–Meier survival curves with log rank tests and Cox proportional hazards models (hazard ratios with 95% CI) were used. Predefined subgroup analyses based on chemotherapy regimen and comorbidities were performed using linear regression models, with treatment by subgroup interaction tests. The interobserver and intraobserver agreement for the measurements of LVEF and LVGLS were analyzed by interclass correlation coefficient. *p *< 0.05 was considered statistically significant.

## Author Contributions

Mei Zhang and Li Li conceived and designed the study. Xiaoling Liu, Mengmeng Li, Wenwen Song, Yu Zhang, Wuyun Bao, Chaoyu Liu, Yuan Zhang and Quande Liu were responsible for patient recruitment, data collection, and follow‐up. Xiaoling Liu, Mengmeng Li, and Wenwen Song analyzed the data and drafted the manuscript. Cheng Zhang, Yun Zhang, Mei Zhang, and Li Li critically revised the manuscript for important intellectual content. All authors have read and approved the final manuscript.

## Funding

This study was supported by the Research Horizontal Projects of Shandong University (No. 301/2021H). The funder had no role in the study design, data collection, analysis, interpretation, or writing of the manuscript.

## Conflicts of Interest

The authors declare no conflicts of interest.

## Ethics Statement

This study was conducted in accordance with the principles of the Declaration of Helsinki and was approved by the Research Ethics Committee of Qilu Hospital, Shandong University (ethics approval number: KYLL‐202008‐191). The trial was registered at ClinicalTrials.gov (NCT05504148). Written informed consent was obtained from all participants.

## Supporting information




**Supporting file 1**: mco270780‐sup‐0001‐SuppMat.docx

## Data Availability

The data that support the findings of this study are available from the corresponding author upon reasonable request.
