# Supplementary material for: Short‐Term, Cycle‐Synchronized Adjunctive Therapy With Crocus Total Glucosides Tablets for Cardiac Protection Against Cancer Therapy‐Related Cardiac Dysfunction in Breast Cancer Patients: A Randomized, Double‐Blind, Placebo‐Controlled Trial
Source: MedComm (2020). 2026 May 28;7(6):e70780. doi: 10.1002/mco2.70780 (PMC13240093; doi:10.1002/mco2.70780)
Supplement: Supplementary file 1 — Supporting file 1: mco270780‐sup‐0001‐SuppMat.docx [file MCO2-7-e70780-s001.docx]

**Short-term, cycle-synchronized adjunctive therapy with Crocus total glucosides tablets for cardiac protection against cancer therapy-related cardiac dysfunction in breast cancer patients: a randomized, double-blind, placebo-controlled trial**

**Running title: Cardiac protection with CTGT in breast cancer**

***By:***

Xiaoling Liu^1#^, MD, PhD, Mengmeng Li^1#^, MD, PhD, Wenwen Song^1,2#^, MD, Yu Zhang^1^, MD, PhD, Wuyun Bao^1,3^, MD, Chaoyu Liu^1^, MD, Yuan Zhang^4,5^, MD, Quande Liu^1^, MD, Cheng Zhang^1^, MD, PhD, Yun Zhang^1^, MD, PhD, Li Li^6^*, MD, PhD, Mei Zhang^1^*, MD, PhD

***From:***

1. State Key Laboratory for Innovation and Transformation of Luobing Theory; Key Laboratory of Cardiovascular Remodeling and Function Research of MOE, NHC, CAMS and Shandong Province; Department of Cardiology, Qilu Hospital of Shandong University, Jinan, China

2. Department of Geriatric Medicine, the Second Hospital, Cheeloo College of Medicine, Shandong University, Jinan, China

3. Department of cardiology, the Second Hospital, Cheeloo College of Medicine, Shandong University, Jinan, China

4. Human Phenome Institute, Zhangjiang Fudan International Innovation Center, Fudan University, Shanghai, China

5. Clinical Epidemiology Unit, Qilu Hospital of Shandong University, Jinan, China

6. Department of Medical Oncology, Qilu Hospital of Shandong University, Jinan, China

^#^ These three authors contributed equally to this study.

***Correspondence Author:**

Mei Zhang, MD, PhD, Department of Cardiology, Qilu Hospital of Shandong University, Jinan, Shandong Province, China, Email: [daixh@vip.sina.com](mailto:daixh@vip.sina.com), or Li Li, MD, PhD, Department of Medical Oncology, Qilu Hospital of Shandong University, Jinan, Shandong Province, China, Email: [drlili5060@163.com](mailto:drlili5060@163.com).

**Lead contact:** Mei Zhang, MD, PhD, Email: [daixh@vip.sina.com](mailto:daixh@vip.sina.com)

**Supplementary Information**

***Supplementary Material 1*:** Detailed materials and methods

***Supplementary Material 2:***Chemotherapy regimens for the patients enrolled in the study

***Supplementary Material 3:*** Detailed information of Crocus total glucosides tablets (CTGT) and placebo (including quality control data)

***Supplementary Material 4:***Reasons for discontinuation

***Supplementary Material 5:***Per‑protocol analysis of primary and secondary outcomes

***Supplementary Material 6:*** *Repeated measures ANOVA assessing LVGLS and LVEF over time*

***Supplementary Material 7:*** *The inter-observer and intra-observer agreement for the measurements of LVEF and LVGLS.*

***Supplementary Material 8:*** *Supplementary* *References*

**Supplementary Material 1: Detailed materials and methods**

***Study design and eligibility***

This study was a double-blind, placebo-controlled randomized clinical trial conducted in Qilu Hospital of Shandong University, China. This was an investigator-initiated trial evaluating a novel, cycle-synchronized regimen of an already-marketed botanical drug, CTGT, for the prevention of CTRCD. The study complies with the principles of the Declaration of Helsinki, and was approved by the Research Ethics Committee of Qilu Hospital, Shandong University (Ethics approval number: KYLL-202008-191) prior to enrollment of the first participant. All participants provided written informed consent. This trial was registered in ClinicalTrials.gov (NCT05504148). Full trial protocol is available from the corresponding author.

***Patients***

Breast cancer patients who were to receive adjuvant chemotherapy and/or adjuvant targeted therapy at Qilu Hospital of Shandong University were recruited from March 2021 to April 2023. The inclusion criteria were as follows: (1) 25-80 years old, female; (2) diagnosed as breast cancer by histopathology; (3) intend to receive adjuvant radiotherapy / chemotherapy or combined with adjuvant trastuzumab or pertuzumab; (4) at least 6 cycles of chemotherapy after enrollment. The exclusion criteria included: (1) pregnant or lactating women; (2) poor image quality of echocardiography that did not allow complete analysis; (3) persistent atrial fibrillation or severe arrhythmia which affect echocardiographic data collection and analysis; (4) participating in other clinical studies of traditional Chinese medicine.

The chemotherapy plan for all the enrolled patients is formulated by the oncologist according to the patient's condition and related treatment guidelines. In short, patients with HER-2 positive breast cancer were treated with A (E) C sequential PH/TH regimen with or without pertuzumab, or TCbH with or without pertuzumab, while patients with HER2 negative breast cancer were treated with A (E) C sequential P/T, or TC regimen. The detailed chemotherapy regimen was shown in the supplementary material 2.

***Randomization and blinding***

Eligible patients were randomly assigned in a 1:1 ratio to receive either CTGT or matching placebo using a stratified block randomization method. A statistician not involved in patient recruitment or outcome assessment generated the random allocation sequence using SAS 14.0 software with a fixed block size of 4. The randomization table (blind bottom) was kept concealed from all study personnel until database lock and sponsor approval at the end of the trial.

Study drugs (CTGT and placebo) were blinded and labelled sequentially according to the randomization numbers. After a patient was deemed eligible, the recruiting researcher dispensed the next available numbered drug pack in sequential order, thereby concealing the allocation from both the patient and the research team. Participants, care providers (oncologists, cardiologists and nurses), and outcome assessors (echocardiographers and laboratory technicians) were all blinded to group assignment throughout the trial. The placebo tablets were identical in appearance, packaging, and taste to CTGT, as detailed in Supplementary Material 3.

***Trial procedure and Investigational Product***

The patients who were confirmed to meet the entry criteria signed the informed consent. After collecting baseline information, the patients were randomly assigned to receive either CTGT or matching placebo, in accordance with the sequestered, fixed-randomization schedule, with the use of balanced blocks to ensure an approximate 1:1 ratio of the two regimens. In each chemotherapy cycle, patients received either CTGT or a matching placebo (4 tablets, 3 times daily) in a brief 8-day regimen, beginning 1 day before chemotherapy and continuing for 8 consecutive days.

CTGT (Reyoung Pharmaceutical Co., Ltd., Zibo, China; batches 21010302 and 21010303) is a standardized botanical drug. Each tablet contains 12 mg of crocus total glucosides, with crocin-1 as the primary marker compound for quality control. According to the national drug standard WS-080(Z-015)-2014Z, each tablet must contain no less than 4.1 mg of crocin-1 and 1.6 mg of crocin-2. The placebo tablets were identical in appearance, packaging, and labeling and were manufactured by the same company (batches 21010302 and 21010303). Quality control for both CTGT and placebo included testing for appearance, content uniformity, disintegration time, and microbial limits, as per the same national standard. Detailed quality control data are provided in the supplementary material 3. The rationale for the 8-day, cycle-synchronized regimen of CTGT is twofold. First, it targets the acute injury window following chemotherapy, as anthracycline-induced cardiotoxicity typically initiates within hours to days [1]. Second, preclinical evidence suggests that crocin does not interfere with, and may even enhance, the antitumor efficacy of chemotherapies like doxorubicin [2,3]. However, given that crocin is a valuable botanical drug and continuous prophylactic use throughout the entire treatment course may be costly and burdensome, we conducted this prospective trial to investigate whether this brief, cycle-synchronized regimen administered only during chemotherapy could offer a more practical and potentially cost-effective cardioprotective strategy.

Adherence was assessed by pill counts at each visit. All patients self-administered the tablets orally. Fidelity was ensured by daily diary and returned blister packs. Patients were followed up and evaluated at 3 months and 6 months, and the follow-up period was 6 months.

***Two-dimensional and Speckle Tracking Echocardiography***

Two-dimensional (2D) echocardiography and speckle tracking echocardiography (STE) were performed during screening, at 3 months and 6 months to assess the cardiac function. Echocardiograms were acquired using the GE vivid E9 and E95 system (GE Healthcare; Vingmed Ultrasound, Horten, Norway) equipped with an M5S probe (1.5-4.6 MHz), according to the guidelines of the American Society of Echocardiography (ASE) by two experienced sonographers. All images were transmitted to the core echo laboratory at Shandong University, where they were reviewed in blinded and independent fashion by two experienced physicians using an EchoPAC (Version.204) workstation, according to American Society of Echocardiography standards. Left ventricular end diastolic (LVEDV) and end systolic volumes (LVESV) were measured in standard apical 4- and 2-chamber views, respectively, with subsequent LVEF calculation using the biplane Simpson's method. Left ventricular strain analysis was conducted on standard apical 2-, 3-, and 4-chamber views. Optimal cardiac cycles were selected with QRS onset as the reference point. All echocardiographic analyses were performed with a frame rate of 40-80 frames per second. End-systole was defined as the point of aortic valve closure (AVC), identified from the apical three-chamber view. Following the standardization protocols recommended by the EACVI/ASE/Industry Task Force [4], the left ventricular endocardium was traced in each apical view at the end-systolic (AVC) frame. Subsequently, the region of interest was automatically generated between the endocardial and epicardial borders and manually adjusted to include the full LV myocardium. LVGLS was then calculated using the software's built-in algorithms.

Thirty subjects were randomly selected by two experienced echocardiographers to assess the consistency of the analyzed results. During the measurement and analysis of the images, the clinical data of the patients and the results of each other's analysis were not visible. The previously analyzed images were measured again by the same echocardiographer 1 month later to assess intra-observer agreement.

***Electrocardiogram and Dynamic Electrocardiogram***

To assess the occurrence of arrhythmias and electrocardiographic changes, standard electrocardiogram was performed during screening, at 3 months and 6 months, while dynamic electrocardiogram were conducted during screening and at 6 months. Severe arrhythmias were defined as any of the following: frequent ventricular premature beats (ventricular premature beats ≥20% of all heart beats), non-persistent or persistent ventricular tachycardia, ventricular fibrillation, sick sinus syndrome, degree II and above atrioventricular block.

***Laboratory Assessment***

The following laboratory indices were analyzed in the laboratory of Qilu Hospital of Shandong University at baseline, 3 months, and 6 months: routine blood, routine urine, fasting blood glucose, alanine aminotransferase (ALT), glutamine aminotransferase, urea nitrogen, creatinine, total cholesterol, triglyceride, low-density lipoprotein cholesterol (LDL-C), high-density lipoprotein cholesterol (HDL-C), international normalized ratio of prothrombin time, active part thrombin time, D-dimer, creatine kinase, creatine kinase isoenzyme MB (CK-MB), and high sensitivity troponin I (hs-cTnI), and N-terminal pro-B type natriuretic peptide (NT-proBNP), potassium (K^+^), sodium (Na^+^), with all of the above indices collected over a 6-hour period in a fasting state.

***Study Endpoints***

The primary endpoints were the relative decline in LVGLS from the baseline and absolute reduction in LVEF percentage points at 6 months. As LVGLS is conventionally reported as a negative value, the relative decline was calculated using absolute values to represent a true deterioration in function: ΔLVGLS% = (|LVGLS_-follow-up_|- |LVGLS_-baseline_|) / |LVGLS_-baseline_| × 100%. Thus, a negative ΔLVGLS% indicates a worsening (decline) in strain, and an absolute value exceeding 15% was considered clinically significant per the CTRCD definition. The absolute reduction in LVEF percentage points was calculated as ΔLVEF = LVEF_-follow-up_-LVEF_-baseline_.

Secondary endpoints included: (1) the relative decline in LVGLS from baseline and absolute reduction in LVEF percentage points at 3 months; (2) LVEF and LVGLS values at both 3-month and 6-month follow-ups; (3) the incidence of CTRCD, defined as either a reduction in LVEF to <50% or a new relative decline in LVGLS by >15% from baseline using absolute values [5], along with the incidence of newly elevated hs-cTnI above the upper limit of normal (ULN) and/or NT-proBNP exceeding the age-specific ULN; and (4) the occurrence of newly emerging severe arrhythmias during the 6-month follow-up period.

Security endpoints included any or more of the following new exceptions: ALT≥3 ULN, serum creatinine level increased by≥30%, digestive tract symptoms such as nausea, vomiting and diarrhea, hemoglobin level ≤ 90g/L, severe bleeding (Bleeding Academic Research Consortium [BARC] grade 3 or above), activity-related chest pain and chest tightness. Adverse events were graded according to the Common Terminology Criteria for Adverse Events (CTCAE) version 5.0. [6]

***Sample Size Analysis***

Sample size estimation was performed using PASS 11.0.7 software with a two-sided α of 0.05 and 80% power (β=0.2), assuming a 1:1 allocation between CTGT and placebo groups. Based on preliminary data showing a (3.28±5.38) % LVEF reduction in untreated controls versus an anticipated ≤ 0.17% reduction in the CTGT group after 6 months, we calculated a required sample size of 48 pairs. To account for potential 20% dropout, 120 patients were ultimately enrolled. No interim analyses were planned or conducted, and there were no stopping guidelines. While our observational data (n=79) demonstrated progressive LVGLS deterioration during chemotherapy [baseline: (-22.6±1.77)%; post-6 cycles: (-16.96±3.65)%; post-8 cycles: (-15.94±4.03)%], the primary sample size calculation relied on LVEF reduction due to insufficient published data regarding crocin's effects on LVGLS preservation during cancer therapy. This may have resulted in the study being underpowered for the LVGLS-based primary endpoint, a limitation that is considered in the discussion.

***Statistical Analysis***

Data were analyzed using SPSS Version 25.0 for Windows (IBM, Armonk, New York) and R software version 4.4.1 (R Foundation for Statistical Computing, Vienna, Austria). All data were tested for normality and homogeneity of variance. Continuous variables conforming to normal distribution were expressed as mean ±SD when conforming to normal distribution, otherwise expressed as the median (interquartile range) [M (Q1, Q3)], and independent samples t-test was used to compare two groups. Categorical variables were expressed as percentages and analyzed using the Chi-square test. The ITT principle was followed for all primary and secondary efficacy analyses, including all 120 randomized participants. Missing data were handled by multiple imputation with chained equations (MICE) under the missing-at-random (MAR) assumption. The imputation model included all available outcome measures (LVEF, LVGLS, hs-cTnI, etc.) at 3 months, and 6 months. Fifty imputed datasets were generated to ensure stability of the imputations. Efficacy analyses were conducted in each imputed dataset and pooled using Rubin's rules to obtain combined estimates, standard errors, and p-values. For the primary and secondary efficacy analyses, continuous outcomes were compared between the two groups using independent samples t-tests, and categorical outcomes were analyzed using the Chi-square test. To further assess the longitudinal trajectories of continuous variables (LVEF and LVGLS) across different time points (baseline, 3 months, and 6 months), a repeated measures analysis of variance (RM-ANOVA) was employed, followed by Bonferroni-corrected post-hoc tests. To control the family-wise error rate for the two co-primary endpoints (ΔLVGLS% and ΔLVEF at 6 months), a Hochberg procedure was applied to the pooled p-values. This procedure tests the larger p‑value against α = 0.05 and, if not significant, tests the smaller p‑value against α/2 = 0.025, thereby preserving an overall Type I error rate of 0.05. Secondary endpoints were analyzed without multiplicity adjustment.

For time-to-event outcomes (CTRCD, ΔLVGLS%>15%, hs-cTnI elevation), Kaplan-Meier survival curves were constructed for the ITT population using the original data (without imputation) as these outcomes are event-based and censoring handles missing data appropriately. Between-group comparisons were performed using the log-rank test, and HR with 95% CI were estimated using a Cox proportional hazards model. To evaluate potential heterogeneity in treatment effect and pre-existing comorbidities, predefined subgroup analyses were performed based on clinically relevant factors, including chemotherapy regimen and comorbidities. For each subgroup, the treatment effect on the primary endpoint (ΔLVEF and ΔLVGLS% at 6 months) was estimated using a linear regression model on each imputed dataset, with the treatment difference expressed as B value with 95% CI pooled across imputations. To assess whether the treatment effect varied across subgroups, treatment-by-subgroup interaction terms were tested by including the interaction term in the regression model on each imputed dataset and then pooling the interaction test results. The inter-observer and intra-observer agreement for the measurements of LVEF and LVGLS were analyzed by interclass correlation coefficient (ICC). *P*<0.05 was considered statistically significant.

**Supplementary Material 2: The chemotherapy regimens for the patients enrolled in the study**

The chemotherapy plan for all the enrolled patient is formulated by the oncologist according to the patient's condition and related treatment guidelines [7].

**2.1 For patients with HER-2 positive breast cancer (HER-2+), the chemotherapy regimen was A(E)C→PH/TH ± Pertuzumab regimen or TCbH ± Pertuzumab regimen, as follows:**

(1) A(E)C→PH/TH ± Pertuzumab regimen: Doxorubicin (A) 50–60 mg/m^2^ on day 1 or Epirubicin (E) 80–100 mg/m^2^ on day 1, Cyclophosphamide (C) 600 mg/m^2^ on day 1, 21 days for a cycle, a total of four cycles;

The sequential schemes A/B are as follows:

A: followed by Paclitaxel (P) 80 mg/m^2^ on the first day, once a week for 12 weeks. At the same time, Trastuzumab (H) was given at the first dose of 4 mg/kg, followed by 2 mg/kg, once a week for 12 weeks. Combined with Pertuzumab, first dose of 840 mg and then 420 mg for each period of 21 days, for a period of four cycles.

B: followed by Docetaxel (T) 100 mg/m^2^ on the first day, 21 days as a cycle, a total of four cycles. At the same time, Trastuzumab (H) was given at the first dose of 8 mg/kg, followed by 6 mg/kg for a period of 21 days, for a total of four cycles. Combined with Pertuzumab, first dose of 840 mg and then 420 mg for each period of 21 days, for a period of four cycles.

(2) TCbH±Pertuzumab regimen:

Docetaxel (T) 75 mg/m^2^ and Carboplatin (Cb) AUC 6 on the first day, 21 days as a cycle, a total of four cycles. At the same time, Trastuzumab (H) was given at the first dose of 8 mg/kg, followed by 6 mg/kg for a period of 21 days, for a total of six cycles. Combined with Pertuzumab, first dose of 840 mg and then 420 mg for each period of 21 days, for a period of four cycles.

Both A(E)C→PH/TH±Pertuzumab and TCbH±Pertuzumab regimens applied trastuzumab (H) at a dose of 6 mg/kg at the end of chemotherapy, combined with or without patuximab dose of 420mg every 3 weeks for a year.

**2.2 For patients with HER-2 negative breast cancer (HER-2-), the chemotherapy regimen was A(E)C→P/T regimen or TC regimen, as follows:**

(1) A(E)C→P/T regimen: Doxorubicin (A) 60 mg/m^2^ on day 1 or Epirubicin (E) 60–100 mg/m^2^ on day 1, Cyclophosphamide (C) 600 mg/m^2^ on day 1, 21 days for a cycle, a total of four cycles; Docetaxel (T) 100 mg/m^2^ iv on day 1, 21 days as a cycle, a total of four cycles.

(2) TC regimen: Docetaxel (T) 75 mg/m^2^ on day 1 and Carboplatin (Cb) AUC 6 on day 1, 21 days for a cycle, a total of six cycles.

**Supplementary Material 3: Detailed Information of Crocus total glucosides tablets (CTGT) and Placebo**

**3.1 *Quality Control Data for CTGT and Placebo***

CTGT (Reyoung Pharmaceutical Co., Ltd., Zibo, China; batches 21010302 and 21010303) and matching placebo (same manufacturer; batches 21010302 and 21010303) were manufactured in compliance with the national drug standard WS-080(Z-015)-2014Z. Each CTGT tablet contains 12 mg of crocus total glucosides, with crocin-1 as the primary marker compound (approximately three‑quarters of the total glucosides). The quality control results for both batches are presented in Tables S 3.1 and S 3.2.

**Table S 3.1. Quality Control Report for Crocus total glucosides tablets (Batch 21010302 and 21010303)**

| **Test Item** | **Specification**  **(WS-080(Z-015)-2014Z)** | **Batch 21010302** | | **Batch 21010303** |
| --- | --- | --- | --- | --- |
| **Appearance** | Film-coated tablets; after removing coating, orange‑yellow to brownish‑red; slight aroma, pungent taste | | Film-coated, brownish‑red core; slight aroma, pungent taste | Film-coated, brownish‑red core; slight aroma, pungent taste |
| **Content uniformity** | Compliant | Compliant | | Compliant |
| **Disintegration time** | ≤ 60 minutes | 18 minutes | | 18 minutes |
| **Microbial limits** |  |  | |  |
| Total aerobic count | ≤ 10³ CFU/g | < 10 CFU/g | | < 10 CFU/g |
| Total yeasts and moulds | ≤ 10² CFU/g | < 10 CFU/g | | < 10 CFU/g |
| *Escherichia coli* | Not detected | Not detected | | Not detected |
| **Assay** |  |  | |  |
| Crocus total glucosides (as crocin‑1) | 85.0% – 115.0% of labelled amount | 100.4% | | 99.60% |
| Crocin‑1 (C44H6_4_O24) | ≥ 4.1 mg per tablet | 5.757 mg | | 5.627 mg |
| Crocin‑2 (C35H52O19) | ≥ 1.6 mg per tablet | 2.258 mg | | 2.232 mg |
| **Conclusion** | Conforms to WS-080(Z-015)-2014Z | Conforms | | Conforms |

**Table S 3.2 Quality Control Report for Placebo Tablets (Batch 21010302 and 21010303)**

| **Test Item** | **Specification (referring to WS-080(Z-015)-2014Z)** | **Batch 21010302** | **Batch 21010303** |
| --- | --- | --- | --- |
| **Microbial limits** |  |  |  |
| Total aerobic count | ≤ 10³ CFU/g | < 10 CFU/g | < 10 CFU/g |
| Total yeasts and moulds | ≤ 10² CFU/g | < 10 CFU/g | < 10 CFU/g |
| *Escherichia coli* | Not detected | Not detected | Not detected |
| **Conclusion** | Conforms to the specified limits | Conforms | Conforms |

Placebo tablets were identical in appearance, packaging, and labelling to the active CTGT tablets.

**3.2 Cost Comparison of CTGT Regimens: 8‑Day/Cycle versus Continuous Daily Prophylaxis**

CTGT is priced at ¥396 per box, with each box containing 24 tablets (12 mg per tablet). The standard daily dose is 12 tablets (4 tablets three times daily), equivalent to 0.5 box per day. Based on this dosing schedule, the 8‑day per cycle regimen requires 4 boxes per cycle (8 days × 0.5 box/day), resulting in a cost of ¥1,584 per cycle. In contrast, a continuous daily regimen over a 21‑day cycle would require 10.5 boxes per cycle (21 days × 0.5 box/day), amounting to ¥4,158 per cycle. Weekly costs are ¥528 for the 8‑day regimen and ¥1,386 for the continuous regimen. Over a 6‑month period (approximately 182 days), assuming 8 chemotherapy cycles within that period, the total cost is ¥12,672 for the cycle‑synchronized regimen and ¥36,036 for continuous daily prophylaxis. This comparison highlights the economic advantage of the cycle‑synchronized strategy, as the 8‑day regimen reduces drug cost by approximately 62% per cycle compared to continuous daily prophylaxis. Detailed cost data are presented in Table S3.3.

**Table S 3.3. Cost Comparison of CTGT Regimens**

| **CTGT Regimens** | **cycle-synchronized Regimen**  **(8 days/cycle)** | **Continuous Daily Regimen**  **(21 days/cycle)** |
| --- | --- | --- |
| Cost per cycle (CNY) | 1584 | 4158 |
| Weekly cost (CNY) | 528 | 1386 |
| Total cost for 6 months (CNY) | 12672 | 36036 |

**Supplementary Material 4. Reasons for Discontinuation**

A total of 16 participants (13.3%) discontinued the study drug or were excluded from the per-protocol analysis during the double-blind treatment interval, including 6 (10.0%) in the CTGT group and 10 (16.7%) in the placebo group (Fisher's exact test, *P* = 0.42). Detailed reasons for discontinuation are presented in Table S 4.1and Table S 4.2

**Table S 4.1 Reasons for Discontinuation**

| **Reason for Discontinuation** | **CTGT Group (n=6)** | **Placebo Group (n=10)** | **Related to Study Intervention** | **Related to Outcome Assessment** |
| --- | --- | --- | --- | --- |
| Withdrawal by participants / refusal of follow-up assessment | 3 | 7 | No | Yes |
| Loss to follow-up (unable to contact) | 2 | 1 | No | Yes |
| Inability to perform TTE^†^ | 0 | 1 | No‡ | Yes |
| Excluded post-hoc (poor data quality; all assessments completed) | 1 | 0 | No | No§ |
| Total | 6 | 10 |  |  |

CTGT, crocus total glucosides tablets; TTE, transthoracic echocardiography. ^†^Radiotherapy-induced chest wall skin damage, anemia, and COVID-19 infection precluded TTE performance. ^‡^The inability to perform echocardiography was attributable to clinical factors unrelated to study drug. ^§^This participant (ID: 002, CTGT group) completed all scheduled assessments but was excluded post-hoc due to poor data quality; consequently, she was classified as an exclusion rather than a dropout in the CONSORT flow diagram.

**Table S 4.2 Individual-Level Discontinuation Details**

| **Patient ID** | **Group** | **Category** | **Reason** |
| --- | --- | --- | --- |
| 003 | CTGT | Withdrawal | Patient misunderstood study procedures; refused to continue |
| 041 | CTGT | Loss to follow-up | Relocated from Shandong to Tianjin after surgery |
| 102 | CTGT | Loss to follow-up | Returned to home province; became unreachable |
| 109 | CTGT | Withdrawal | Transferred to another hospital; declined return |
| 120 | CTGT | Withdrawal | Declined study examinations and medication from baseline |
| 002 | CTGT | Exclusion | Completed all assessments; excluded due to poor data quality |
| 008 | Placebo | Withdrawal | Returned to home province; declined further assessments |
| 014 | Placebo | Withdrawal | Refused post-surgical follow-up assessments |
| 020 | Placebo | Withdrawal | Declined to return for follow-up |
| 036 | Placebo | Withdrawal | Declined to return for follow-up |
| 053 | Placebo | Withdrawal | Declined to return for follow-up |
| 081 | Placebo | Withdrawal | Refused follow-up assessment |
| 082 | Placebo | Withdrawal | Refused follow-up assessment |
| 094 | Placebo | Loss to follow-up | Transferred to another institution; all contact attempts failed |
| 103 | Placebo | Unable to complete TTE | Chest wall skin damage from radiotherapy; comorbid anemia and COVID-19 |
| 105 | Placebo | Withdrawal | Transferred to another hospital; self-discontinued study medication |

**Supplementary Material 5. Per-Protocol Analysis of Primary and Secondary Outcomes**

**5.1 *Statistical analysis for per-protocol population***

Data were analyzed using SPSS Version 25.0 for Windows (IBM, Armonk, New York). All data were tested for normality and homogeneity of variance. Continuous variables conforming to normal distribution were expressed as mean ± SD, otherwise expressed as median with interquartile range [M (Q1, Q3)]. Independent sample t-test was used for comparison of normally distributed continuous variables between two groups, and the Mann-Whitney U test was applied for non-normally distributed variables. Categorical variables were expressed as counts and percentages and compared using the Chi-square test or Fisher's exact test, as appropriate.

The per-protocol analysis included all participants who completed the 6-month follow-up without major protocol deviations. For continuous outcomes (LVEF, LVGLS, ΔLVEF, ΔLVGLS%), between-group comparisons were performed using independent sample t-test for normally distributed data or Mann-Whitney U test for non-normally distributed data. Within-group changes from baseline were assessed using paired t-test or Wilcoxon signed-rank test, as appropriate.

For time-to-event outcomes (CTRCD, ΔLVGLS% >15%, hs-cTnI elevation), Kaplan-Meier survival curves were constructed for the per-protocol population. Between-group comparisons were performed using the log-rank test. A two-sided *P* value < 0.05 was considered statistically significant.

**5.2 Supplementary Results of Per-Protocol Analysis**

A total of 104 participants, including 54 assigned to CTGT and 50 assigned to matching placebo, completed the 6-month follow-up and were included in the per-protocol analysis. Baseline characteristics of these participants remained well-balanced between groups (data not shown).

***5.2.1 Primary Outcomes***

At the 6-month follow-up, the CTGT group demonstrated a significantly smaller relative decline in LVGLS from baseline compared to the placebo group [−5.11% (−11.65% to 2.85%) vs. −11.80% (−20.52% to −4.90%), *P* = 0.003] (Supplementary Table I). In contrast, the reduction in LVEF percentage points did not differ significantly between groups (CTGT group: −4.13 ± 5.52% vs. placebo group: −2.44 ± 5.49%, *P* = 0.129).

***5.2.2 Secondary Outcomes***

At the 3-month follow-up, the CTGT group exhibited a significantly smaller relative decline in LVGLS from baseline compared to the placebo group [−2.71% (−7.36% to 2.28%) vs. −9.03% (−15.36% to −4.75%), *P* = 0.002]. The reduction in LVEF percentage points at 3 months did not differ significantly between groups (−2.19 ± 4.38% vs. −1.24 ± 3.99%, *P* = 0.274) (Table S5).

At both the 3-month and 6-month follow-ups, LVGLS values were significantly better (more negative) in the CTGT group compared to the placebo group (*P* = 0.039 and *P* = 0.033, respectively). No significant differences were observed in LVEF values between groups at either time point (both *P* > 0.05) (Table S5).

During the 6-month follow-up, a new relative decline in LVGLS >15% occurred in significantly fewer participants in the CTGT group compared to the placebo group [8 (14.8%) vs. 17 (34.0%), *P* = 0.022]. No participants in either group developed LVEF decline to <50%. New hs-cTnI elevations (>ULN) were observed in 2 patients (3.7%) in the CTGT group and 4 (8.0%) in the placebo group (*P* = 0.348). Neither group exhibited new NT-proBNP elevations above the age-specific ULN or new-onset severe arrhythmias. The incidence of CTRCD was significantly lower in the CTGT group compared with the placebo group [10 (18.5%) vs. 21 (42.0%), *P* = 0.009] (Table S5).

**Table S5. Primary and secondary cardiovascular outcomes (per-protocol population).**

| **Parameters** | **CTGT group (n=54)** | **Placebo group (n=50)** | ***P* value** |
| --- | --- | --- | --- |
| **Primary outcome event** |  |  |  |
| Reduced percentage points of LVEF at 6 months (%) | -4.13±5.52 | -2.44±5.49 | 0.129 |
| Relative decline in LVGLS at 6 months (%) | -5.11(-11.65, 2.85) | -11.80(-20.52, -4.90) | 0.003 |
| **Secondary outcome event** |  |  |  |
| Reduced percentage points of LVEF at 3 months (%) | -2.19±4.38 | -1.24±3.99 | 0.274 |
| Relative decline in LVGLS at 3 months (%) | -2.71(-7.36, 2.28) | -9.03(-15.36, -4.75) | 0.002 |
| LVEF at 3 months (%) | 63.44±3.75 | 63.04±4.74 | 0.641 |
| LVGLS at 3 months (%) | -20.16±2.10 | -18.96±3.24 | 0.039 |
| LVEF at 6 months (%) | 61.48±4.66 | 61.80±5.15 | 0.747 |
| LVGLS at 6 months (%) | -20.52±2.35 | -18.34±2.80 | 0.033 |
| LVEF decline to <50% (n, %) | 0(0) | 0(0) |  |
| New relative decline in LVGLS by >15% from baseline (n, %) | 8(14.8%) | 17(34.0%) | 0.022 |
| hs-cTnI > ULN (n, %) | 2(3.7%) | 4(8.0%) | 0.348 |
| NT-proBNP > ULN of the corresponding age group (n, %) | 0(0) | 0(0) | - |
| Newly emerging severe arrhythmias (n, %) | 0(0) | 0(0) | - |
| Incidence of CTRCD | 10(18.5%) | 21 (42.0%) | 0.009 |

Continuous variables conforming to normal distribution are expressed as mean ± SD; non-normally distributed variables are expressed as median (interquartile range). CTGT, crocus total glucosides tablets; CTRCD, cancer therapy-related cardiac dysfunction; LVEF, left ventricular ejection fraction; LVGLS, left ventricular global longitudinal strain; hs-cTnI, high-sensitivity cardiac troponin I; NT-proBNP, N-terminal pro-B type natriuretic peptide; ULN, upper limit of normal.

Kaplan-Meier analysis demonstrated a significantly higher CTRCD-free survival probability in the CTGT group compared to the placebo group (log-rank *P* = 0.001; Figure S5 A). Significant differences in survival probability were also observed for ΔLVGLS% >15% (log-rank *P* = 0.0015; Figure S5 B), whereas no significant difference was observed for cTnI elevation (log-rank *P* = 0.347; Figure S1C).

**
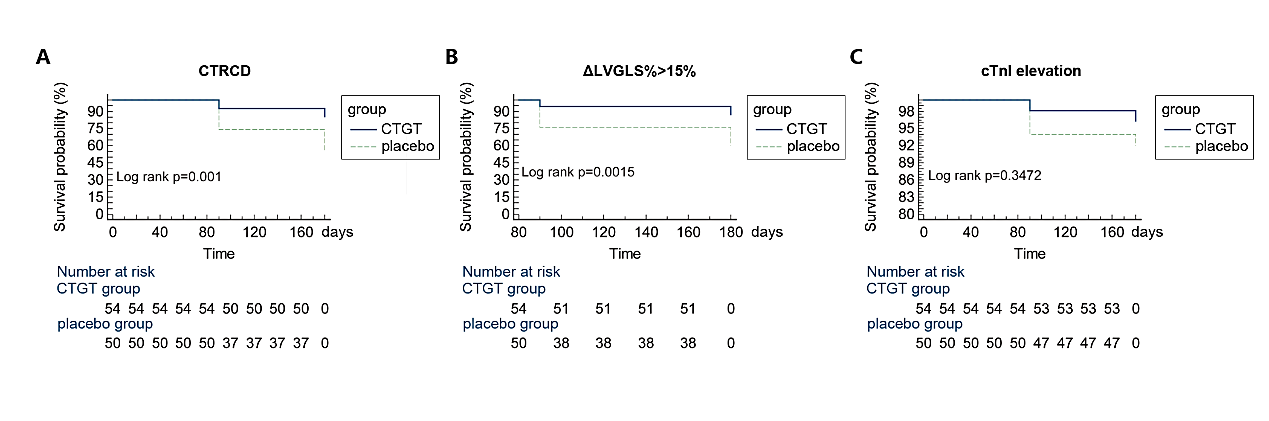
**

**Figure S5. Kaplan–Meier survival curves for CTRCD, ΔLVGLS% and cTnI elevation in the placebo group and CTGT group (per-protocol population).**

Kaplan–Meier curves showing cumulative survival probability for **(A)** CTRCD, **(B)** relative decline in LVGLS >15%, and **(C)** cTnI elevation in the placebo (dashed green line, n=50) and CTGT (solid blue line, n=54) groups. Survival was significantly higher in the CTGT group for CTRCD (log-rank *P* = 0.001) and ΔLVGLS% >15% (log-rank *P* = 0.0015), but not for cTnI elevation (*P* = 0.347). CTGT, crocus total glucosides tablets; cTnI, cardiac troponin I; CTRCD, cancer therapy-related cardiac dysfunction; ΔLVGLS%, relative decline in left ventricular global longitudinal strain from baseline.

**Supplementary Material 6. Repeated measures ANOVA assessing LVGLS and LVEF over time**

**Table S 6.1 Repeated measures ANOVA assessing LVGLS over time**

| **Parameters** | **CTGT group**  **(n=60)** | **Placebo group**  **(n=60)** | ***P* value** |
| --- | --- | --- | --- |
| **LVGLS at baseline (%)** | -20.77±2.37 | -21.28±3.21 | 0.334 |
| **LVGLS at 3 months (%)** | -19.86±2.34* | -19.59±3.35* | 0.611 |
| **LVGLS at 6 months (%)** | -19.30±2.36*^#^ | -18.51±3.36*^#^ | 0.142 |

F_Group_=0.149, P_Group_=0.700; F_Time_=43.406, P_Time_<0.001; F_Group×Time_=4.123, P_Group×Time_=0.019. *Compared with baseline within the same group, ^#^Compared with 3 months within the same group. CTGT, crocus total glucosides tablets; LVGLS, left ventricular global longitudinal strain.

**Table S 6.2 Repeated measures ANOVA assessing LVEF over time**

| **Parameters** | **CTGT group**  **(n=60)** | **Placebo group**  **(n=60)** | ***P* value** |
| --- | --- | --- | --- |
| **LVEF at baseline (%)** | 65.70±4.23 | 64.23±4.50 | 0.070 |
| **LVEF at 3 months (%)** | 63.53±3.81* | 63.02±4.64* | 0.518 |
| **LVEF at 6 months (%)** | 62.03±5.29*^#^ | 61.83±5.14*^#^ | 0.839 |

F_Group_=1.095, P_Group_=0.298; F_Time_=17.682, P_Time_<0.001; F_Group×Time_=0.988, P_Group×Time_=0.375. *Compared with baseline within the same group, ^#^Compared with 3 months within the same group. CTGT, crocus total glucosides tablets; LVEF, left ventricular ejection fraction.

**Supplementary Material 7. The inter-observer and intra-observer agreement for the measurements of LVEF and LVGLS.**


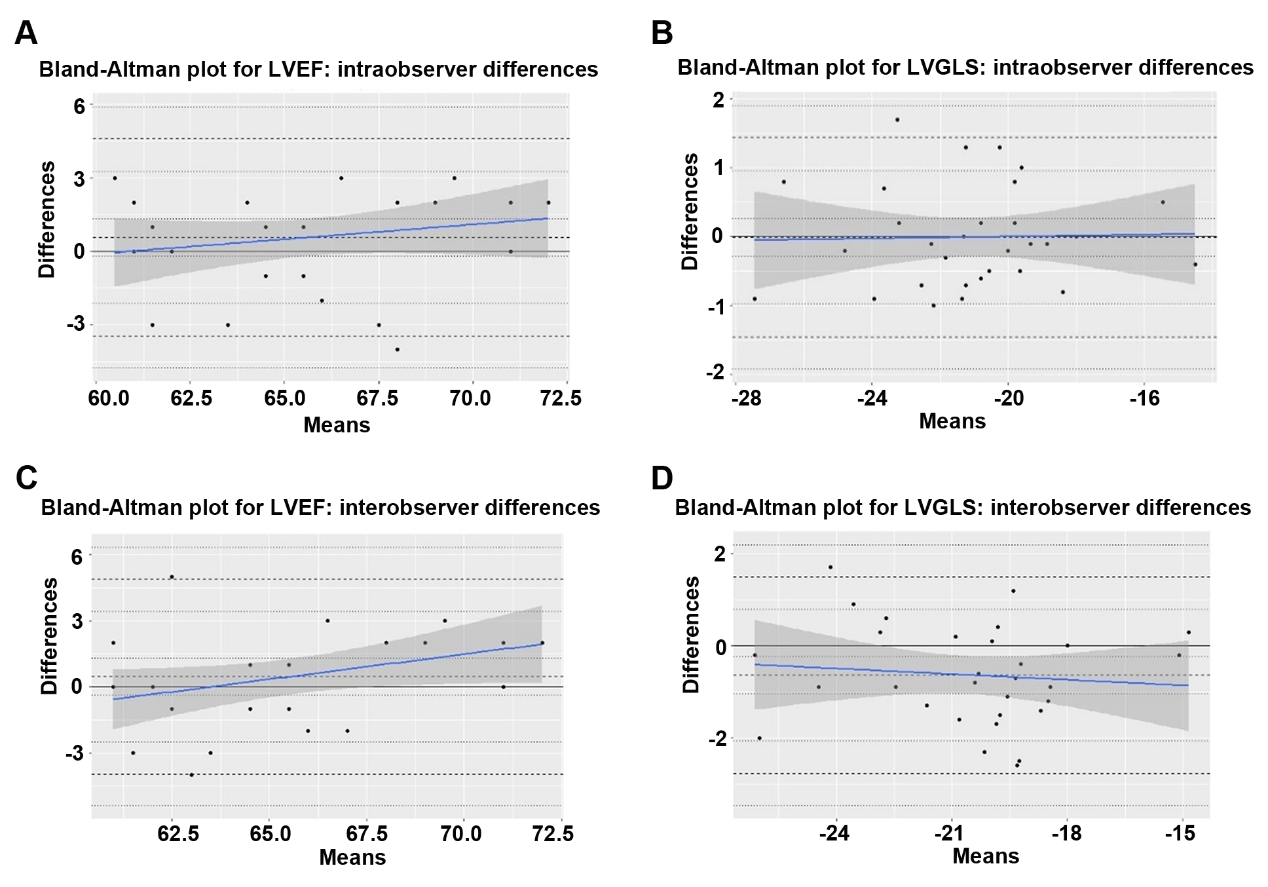


**Figure S7. Bland-Altman analysis of intra- and interobserver differences for LVEF and LVGLS.**

Intraobserver and interobserver variability for LVEF (A, C) and LVGLS (B, D) done by the first observer 4 weeks apart and between the measurements made by two observers. L Bland-Altman plots show good agreement between measurements. LVEF, left ventricular ejection fraction; LVGLS, left ventricular global longitudinal strain.

**Supplementary Material 8. Supplementary References**

1. C. Y. Kong, Z. Guo, P. Song, et al., "Underlying the Mechanisms of Doxorubicin-Induced Acute Cardiotoxicity: Oxidative Stress and Cell Death," *Int J Biol Sci* 18, no. 2 (2022): 760-770.

2. M. A. Hussain, N. M. Abogresha, G. AbdelKader, et al., "Antioxidant and Anti-Inflammatory Effects of Crocin Ameliorate Doxorubicin-Induced Nephrotoxicity in Rats," *Oxid Med Cell Longev* 2021 (2021): 8841726.

3. E. Altinoz, D. Cetinavci, S. A. Abdulkareem Aljumaily, et al., "Crocin, the compound of the dried stigma of Crocus sativus L (saffron), restores doxorubicin-induced disturbances in kidney functioning, oxidative stress, inflammation, renal tissue morphology and TGF-β signalling pathways," *Nat Prod Res* 39, no. 16 (2025): 4609-4622.

4. J. U. Voigt, G. Pedrizzetti, P. Lysyansky, et al., "Definitions for a common standard for 2D speckle tracking echocardiography: consensus document of the EACVI/ASE/Industry Task Force to standardize deformation imaging," *J Am Soc Echocardiogr* 28, no. 2 (2015): 183-193.

5. A. R. Lyon, T. López-Fernández, L. S. Couch, et al., "2022 ESC Guidelines on cardio-oncology developed in collaboration with the European Hematology Association (EHA), the European Society for Therapeutic Radiology and Oncology (ESTRO) and the International Cardio-Oncology Society (IC-OS)," *Eur Heart J* 43, no. 41 (2022): 4229-4361.

6. National Cancer Institute. 2017. “Common Terminology Criteria for Adverse Events (CTCAE) Version 5.0.” U.S. Department of Health and Human Services. https://ctep.cancer.gov/protocoldevelopment/electronic_applications/ctc.htm#ctc_50.

7. H. J. Burstein, G. Curigliano, S. Loibl, et al., "Estimating the benefits of therapy for early-stage breast cancer: the St. Gallen International Consensus Guidelines for the primary therapy of early breast cancer 2019," *Annals of Oncology* 30, no. 10 (2019): 1541-1557.
